# Liposomal Delivery of *Olea europaea* L. Leaf Polyphenols: From Extraction to Functional Evaluation in a Hyperglycemia Cell Model

**DOI:** 10.3390/pharmaceutics18080965

**Published:** 2026-08-06

**Authors:** Immacolata Faraone, Maria Ponticelli, Simona Demuro, Antonio Vassallo, Margherita Accardo, Ludovica Lela, Carla Caddeo, Luigi Milella

**Affiliations:** 1 Department of Health Sciences, University of Basilicata, Via dell’Ateneo Lucano 10, 85100 Potenza, Italy; immacolata.faraone@unibas.it (I.F.); maria.ponticelli@unibas.it (M.P.); ludovica.lela@unibas.it (L.L.); luigi.milella@unibas.it (L.M.); 2 Department of Life and Environmental Sciences, University of Cagliari, 09042 Monserrato, Cagliari, Italy; simona.demuro@unica.it (S.D.); caddeoc@unica.it (C.C.)

**Keywords:** olive leaf polyphenols, phenolic extraction optimization, LC–MS metabolomics, liposomes, gastrointestinal delivery, nutraceutical/pharmaceutical nanoformulation, antioxidant/antihyperglycemic activity

## Abstract

**Background/Objectives**: Olive leaf polyphenols exhibit strong antioxidant and antidiabetic potential. However, their application in nutraceutical and pharmaceutical products is hindered by limited stability, poor solubility, and susceptibility to gastrointestinal degradation. Liposomes offer a viable strategy to enhance their protection and functional performance. **Methods**: A formulation-driven strategy was employed to develop a gastro-resistant liposomal system for olive leaf polyphenols. Extraction conditions were optimized using a Box–Behnken response surface design to maximize phenolic recovery while ensuring compatibility with phospholipid-based systems. Antioxidant activity and key secoiridoids were assessed by spectrophotometric assays and LC–MS/MS. The optimized extract was incorporated into uncoated and Eudragit^®^ L100-coated liposomes, which were physicochemically characterized. Antidiabetic effects were evaluated in intestinal STC-1 cells under glucose-induced hyperglycemic conditions. **Results**: The optimized extract (OE) exhibited high antioxidant activity (124.56 ± 9.57 mg GAE/g, 151.61 ± 2.77 mg TE/g, and 472.92 ± 26.14 mg TE/g in TPC, DPPH and FRAP assays, respectively). LC-HRMS metabolomic profiling confirmed a balanced phytochemical composition, with oleuropein as the main compound (143.144 ± 4.914 mg/g). The optimized Eudragit^®^-coated liposomes were spherical unilamellar vesicles with a mean diameter of 101 ± 6.1 nm, moderate polydispersity (0.46 ± 0.02), and a negative zeta potential (−17 ± 3.5 mV). High entrapment efficiency was achieved, reaching 65 ± 2.1% for oleuropein and 96 ± 0.3% for hydroxytyrosol. The structural integrity of the vesicles was maintained during storage and in the simulated gastrointestinal environment. The nanoformulation reduced intestinal glucose uptake and intracellular reactive oxygen species levels, and restored GLP-1 levels. **Conclusions**: The combination of optimized extraction and liposome-based formulation enabled the development of stable, delivery-ready olive leaf polyphenols for potential nutraceutical and pharmaceutical applications.

## 1. Introduction

Oxidative stress is a key contributor to the development of type 2 diabetes mellitus (T2DM), a metabolic disorder marked by persistent hyperglycemia resulting from impaired insulin secretion by pancreatic *beta* cells and increased peripheral insulin resistance [1]. *Beta* cells progressively lose their ability to produce and release insulin due to oxidative stress, chronic inflammation, and intracellular lipid accumulation, while skeletal muscle, liver, and adipose tissue show reduced insulin sensitivity, impairing glucose uptake [2]. This dual dysfunction alters glycemic homeostasis, promoting metabolic and cardiovascular complications [3]. Given the fundamental role of oxidative stress and chronic inflammation in T2DM pathogenesis, the use of bioactive compounds capable of modulating these processes represents an approach of growing scientific interest.

Italy is a prominent producer of olive oil within the European Union, and its agricultural supply chain annually produces millions of tons of residues, such as pruning waste and olive leaves. Recent estimates indicate that approximately 5.5–6.5 million tons of olive-derived residues are produced annually, including 1.65–3.30 million tons of pruning residues and about 185–231 thousand tons of leaves and small branches generated during olive harvesting and oil processing [4]. In particular, the present study focuses on Ogliarola del Vulture, a cultivar primarily grown in the Basilicata region, particularly in the Vulture, Colline Materane, and Medio Agri Basento areas, and to a lesser extent in some municipalities of the Province of Foggia (the Capitanata region); it is also expanding further into areas of central and southern Italy. It is a variety with medium to high vigor, featuring a medium to dense canopy with a spreading habit [5]. The leaves are elliptical-lanceolate, of medium length, broad, with a light green upper surface. The inflorescence is of medium length, sparse, and bears a relatively low number of flowers. The fruit is a small drupe (1.8 g), ovoid, asymmetrical, with a rounded apex and a truncated base. The variety exhibits a low self-fertility index. The oil produced from this cultivar has an organoleptic profile characterized by moderate levels of fruitiness, bitterness, and pungency, with moderate notes of almond, grass, and artichoke [5]. According to Article 7 of the Production Specification for the Protected Designation of Origin (PDO) “Vulture” [6], the geographical area designated for the Ogliarola cultivar is Monte Vulture, an extinct volcano located in the central part of the southern Apennines, about 60 km from the sea. As a consequence, the soils of the Vulture region are rich in exchangeable potassium (averaging over 450 ppm), exchangeable calcium (averaging over 3000 ppm), and exchangeable magnesium (averaging over 170 ppm). The olive groves used to produce Vulture olive oil are located on the east- and southeast-facing slopes of Monte Vulture, as the mountain influences the microclimate and protects the groves from cold winter winds. The designated area extends across an elevation range of 400 to 700 m above sea level and has a distinctive microclimate characterized by continental-type conditions, with generally long, cold winters and short, often dry summers [6]. In accordance with the regulations set forth in the MASAF Ministerial Guidelines [6], the Ogliarola del Vulture accounts for at least 60–70% of the oil used in the Vulture D.O.P. blend. Other approved cultivars, including Coratina, Leccino, and Frantoio, may account for the remaining 30–40%. In the Basilicata region, approximately 5 million olive trees are cultivated over 28,000 hectares, involving about 30,000 farms and 154 olive mills operating during the annual harvesting and oil-processing season. Olive oil production totals around 6000 metric tons [7]. Hence, the valorization of agro-industrial by-products generated by this cultivation system represents a key strategy for promoting sustainable and circular bioeconomy approaches while providing a valuable source of bioactive compounds.

Among natural bioactive compounds, *Olea europaea* L. leaves, often considered waste from industrial production processes, are a rich source of specialized metabolites, including oleuropein, hydroxytyrosol, tyrosol, and other polyphenols with well-documented health benefits [8]. It was indeed demonstrated that *O. europaea* leaf extracts exhibit significant antioxidant activity and high phenolic content, with oleuropein as the main bioactive constituent; these phenolic compounds confer anti-inflammatory and potentially antimicrobial properties [9].

Furthermore, emerging evidence demonstrates that such polyphenols, and oleuropein in particular, exert an antidiabetic effect, primarily by inhibiting key pro-inflammatory pathways; specifically, they reduce NF-κB transcription and attenuate the secretion of circulating cytokines such as TNF-α [10]. This reduction in low-grade chronic inflammation directly alleviates oxidative stress and prevents the impairment of signaling pathways mediated by the insulin receptor substrate (IRS). Indeed, oleuropein has been shown to increase peripheral glucose uptake in skeletal muscle and adipose tissue and to improve pancreatic β-cell viability, providing a solid biochemical rationale for its protective role against metabolic disorders [11,12].

Given the growing demand for functional and sustainable natural ingredients with proven health benefits, it is necessary to optimize extraction processes to maximize the concentration of specialized metabolites and, consequently, improve the biological activity of the resulting extracts. In this context, ethanol emerges as an extremely advantageous solvent, being environmentally friendly, biodegradable, and safe for nutraceutical/pharmaceutical applications, allowing the efficient extraction of polyphenolic compounds [9]. Simultaneously, these extraction strategies can be integrated into circular economy principles by transforming agricultural waste into high-value-added ingredients, thereby reducing environmental impact and improving waste management efficiency [13].

Polyphenol extraction is greatly influenced by operating conditions, such as time, temperature, and solvent composition [14,15]. The present study aimed to optimize, for the first time, the extraction process of *O. europaea* cv Ogliarola del Vulture leaves by applying the Response Surface Methodology (RSM) based on the Box–Behnken Design (BBD). This approach allowed evaluation of the effects of solvent composition, temperature, and extraction time on the extract’s antioxidant activity and on the amount of characteristic specialized metabolites.

Although optimizing extraction parameters maximizes phenolic yield and antioxidant capacity, this does not translate into maximized *in vivo* activity, especially when the extracts are to be administered orally, due to the intrinsic instability and limited gastrointestinal bioavailability of natural compounds [16,17]. Effective delivery is therefore necessary to preserve the extract’s compounds and increase their bioavailability. Therefore, in this study, nanocarriers were used to incorporate *O. europaea* leaf extract and enhance polyphenols’ stability, bioavailability, and biological activity. Nanocarriers such as liposomes (self-assembled vesicles composed of phospholipids) have indeed been shown to improve the stability, solubility, biodistribution, and therapeutic efficacy of bioactive compounds [18]. Given their amphiphilic structure, these vesicles can simultaneously carry extract’s hydrophilic and hydrophobic compounds, and enable controlled release and targeted distribution while minimizing toxicity. Liposomes are highly versatile and adaptable delivery vehicles [19]. Their surface can be functionalized through various chemical, non-covalent, and biological modifications, such as polyethylene glycol coating (PEGylation), ligand decoration, stimuli-responsive and biomimetic coatings to enhance stability, prolong circulation time, and actively target specific diseased cells [20].

If not properly designed, liposomes can be destabilized under gastric conditions: low pH, pepsin digestion, and mechanical shear can cause aggregation, fusion, or structural breakage leading to premature leakage or degradation of the payload [21]. Therefore, enteric polymers, such as Eudragit^®^, are being used to coat liposomes for oral delivery. The Eudragit^®^ coating provides additional protection to the liposomes’ cargo via pH-sensitivity by remaining insoluble in the acidic environment of the stomach and dissolving in the higher pH environment of the small intestine. This ensures that the optimized extract maintains its functional activity during gastrointestinal transit.

In conclusion, the present study aimed to obtain an optimized extract from *O. europaea* leaves with the highest antioxidant activity and the greatest amount of bioactive compounds, with particular attention to oleuropein, hydroxytyrosol, and tyrosol. This study further aimed to evaluate the incorporation of optimized extract into gastro-resistant liposomal formulation to improve stability, bioavailability and efficacy under hyperglycemic conditions characteristic of diabetes mellitus.

## 2. Materials and Methods

### 2.1. Chemicals

The reagents 2,4,6-tripyridyl-*s*-triazina (TPTZ), ferric chloride (FeCl_3_·6H_2_O), Folin–Ciocalteu, sodium carbonate (Na_2_CO_3_), 2,2-Diphenyl-1-picrylhydrazyl (DPPH) radical, 6-hydroxy-2,5,7,8-tetramethylchroman-2-carboxylic acid (Trolox), absolute ethanol, gallic acid, analytical standards including oleuropein, tyrosol, hydroxytyrosol, the solvents used to prepare samples and for LC-HRMS analysis were purchased from Sigma-Aldrich/Merck (Milan, Italy). Methanol was obtained as LC gradient grade. Distilled water was obtained using a Milli-Q RG purification system (Millipore, Burlington, MA, USA).

Liposomes were prepared using Phospholipon^®^ 90G (P90G) and 1,2-Dioleoyloxy-3-trimethylammonium propane chloride (DOTAP) purchased from Lipoid GmbH (Ludwigshafen, Germany), Eudragit^®^ L100 supplied by Evonik Industries AG (Essen, Germany), and Tween80 and phosphate-buffered saline (PBS) purchased from Sigma-Aldrich/Merck (Milan, Italy).

Reagents for cell culture were purchased from Euroclone (Milan, Italy). 3-[4,5-dimethylthiazol-2-yl]-2,5 diphenyl tetrazolium bromide (MTT), 2′,7′-Dichlorodihydrofluorescein diacetate (DCFH-DA), *N*-acetyl-L-cysteine (NAC) and 2-(*N*-(7-nitrobenz-2-oxa-1,3-diazol-4-yl)amino)-2-deoxyglucose (2-NBDG) were purchased from Sigma–Aldrich/Merck (Milan, Italy).

### 2.2. Plant Material

Leaves of *O. europaea* cv Ogliarola del Vulture were collected as waste of routine pruning operations in January 2025 in Boreano plain, in the municipality of Venosa (Potenza, Italy), located on the slopes of Monte Vulture (approximately 40.9628° N, 15.8129° E), at an altitude of approximately 420 m above sea level. Botanical identification was carried out by Professor Luigi Milella, and a voucher specimen (HLUC n. 014574) was deposited at University of Basilicata.

The plant material was air dried to constant weight, after which it was stored at room temperature. Moisture loss was calculated according to Equation (1).

(1) $$\%\;Moisture\;loss=\left(\frac{initial\;weight-final\;weight}{initial\;weight}\right)\times 100$$

The dried leaves were finely milled to obtain a homogeneous particle size (about 0.5 cm), suitable for downstream extraction processes.

### 2.3. Extraction Procedure

The extraction of dried, micronized *O. europaea* leaves was optimized according to the Response Surface Methodology (RSM) based on the Box–Behnken Design (BBD) with three levels and three factors. These factors (solvent composition, temperature, and time) were selected based on prior studies [14,22] and in-house experiments and used to obtain an extract optimized in terms of antioxidant activity (evaluated as scavenging and reducing activity with the DPPH and the FRAP assay, respectively), total phenolic content (measured with the TPC assay), and the amount of tyrosol, hydroxytyrosol, and oleuropein (identified and quantified via LC-HRMS).

The experimental design developed using Design-Expert v.13 software (Stat-Ease Inc., Minneapolis, MN, USA), consisted of 17 experimental runs, including five center points. A constant 1:10 plant-to-solvent ratio was applied for all extracts. The levels used in the design, both coded and actual, are reported in Table 1.

Temperature was precisely controlled using a thermostatically regulated water bath (Memmert WNB 14, Memmert GmbH + Co. KG, Schwabach, Germany). All extractions were carried out in triplicate under continuous stirring. At the end of the process, the mixtures were filtered through filter paper, and the filtrates were evaporated to dryness under reduced pressure using a rotary evaporator. The resulting extracts were stored at room temperature in the dark until further use.

For each experimental run, the extraction yield was calculated as the percentage ratio between the dry extract obtained after solvent evaporation and the initial dry plant material employed for extraction. These data provide an overall mass balance of the extraction process and allow evaluation of the practical applicability of the optimized conditions.

The influence of the independent variables on the corresponding responses was assessed using a second-order polynomial model (Equation (2)).

(2) $$Y\;=\;\beta_{0}\;+\sum_{i=1}^{3}\beta_{i}X_{i}+\sum_{i=1}^{3}\beta_{ii}X_{i}^{2}+\sum_{i=1}^{2}\sum_{j=i+1}^{3}\beta_{ij}X_{i}X_{j}$$

where *Y* represents the dependent variable and the intercept regression coefficient is denoted by *β*_0_. The independent variables’ regression coefficients for the linear, quadratic, and interaction terms are *βi*, *βii*, and *βij*, respectively [23].

A multiple regression analysis was then performed to develop a predictive model describing the relationship among the studied factors. The adequacy of the second-order polynomial model was assessed based on the outcomes of the ANOVA analysis, taking into account parameters such as the Lack of Fit, the coefficient of determination (R^2^), the adequate precision, and the coefficient of variance (CV%). For each fitted model, contour and three-dimensional surface plots were generated to illustrate the relationships between the experimental factors and the corresponding responses.

#### Extraction Optimization

Based on the BBD model outcomes, the optimization procedure was performed using the numerical optimization function implemented in Design-Expert v.13 software (Stat-Ease Inc., Minneapolis, MN, USA). The optimized conditions were experimentally reproduced in triplicate to validate the predictive capability of the model. The experimental values obtained for all selected responses (TPC, DPPH, FRAP, hydroxytyrosol, tyrosol, and oleuropein) were compared with the corresponding values predicted by the model to assess its predictive performance. Following this validation, the optimized *O. europaea* leaf extract (OE) was prepared under the validated conditions.

### 2.4. Antioxidant Activity

The antioxidant activity of all samples was determined using three complementary *in vitro* spectrophotometric assays, each investigating different mechanisms and aspects of antioxidant capacity. All measurements were performed in triplicate using a UV–Vis spectrophotometer (SPECTROstar^Nano^ BMG Labtech, Ortenberg, Germany). Results were expressed based on a calibration curve generated with specific assay reference standard.

#### 2.4.1. Determination of Total Phenolic Content (TPC)

The Folin–Ciocalteu colorimetric method was used to determine TPC, as previously reported [24]. Results were expressed as milligrams of Gallic Acid Equivalents per gram of dry extract (mg GAE/g), based on a calibration curve generated with gallic acid as reference standard.

#### 2.4.2. 2,2-Diphenyl-1-Picrylhydrazyl (DPPH) Radical Scavenging Assay

The DPPH radical scavenging assay is among the most widely employed spectrophotometric methods for evaluating the antioxidant potential of bioactive compounds and plant extracts [24]. The stable radical 2,2-diphenyl-1-picrylhydrazyl (DPPH) undergoes reduction in the presence of electron or hydrogen atom donors, resulting in a color change from violet to yellow, which can be quantified spectrophotometrically. The decrease in absorbance is proportional to the extract’s ability to neutralize the radical. Fifty μL of a series of dilutions of either Trolox standard or extract were added to 96-well microplates containing 200 μL of a methanolic DPPH solution (100 μM). The reaction mixtures were incubated for 30 min in the dark at room temperature, after which absorbance was recorded at 515 nm. The reduction in absorbance of the radical solution was used as an indicator of scavenging activity. Results were expressed as milligrams of Trolox Equivalents per gram of dry extract (mg TE/g), based on a calibration curve built using Trolox as reference standard, according to a previously reported method [24].

#### 2.4.3. Ferric Reducing Antioxidant Power (FRAP) Assay

The FRAP assay enables the quantification of the antioxidant capacity of extracts by measuring their ability to reduce ferric ions (Fe^3+^) to ferrous ions (Fe^2+^) under acidic conditions [24]. An amount of 25 μL of extract or Trolox standard was added to 225 μL of FRAP reagent. The latter was prepared by mixing 300 mM acetate buffer (pH 3.6), an aqueous solution of 20 mM FeCl_3_·6H_2_O, and a 10 mM solution of 2,4,6-tripyridyl-*s*-triazine (TPTZ) in 40 mM HCl, in a 10:1:1 (*v*/*v/v*) ratio. The reaction mixtures were incubated at 37 °C for 40 min in the dark, after which absorbance was measured at 593 nm. An increase in absorbance is indicative of a higher reducing capacity of the extract. Trolox was used as reference standard, and results were expressed as milligrams of Trolox Equivalents per gram of dry extract (mg TE/g), according to a previously reported method [24].

### 2.5. LC-HRMS Analysis

All *O. europaea* extracts were analyzed by high-performance liquid chromatography coupled to mass spectrometry in order to determine the content of three characteristic compounds of this plant species, namely oleuropein, tyrosol, and hydroxytyrosol [25].

Oleuropein, tyrosol, and hydroxytyrosol were dissolved in 80% MeOH at concentrations ranging from 0.1 to 200 ppm. Two independent biological replicates were prepared, each analyzed in triplicate.

The extracts were dissolved at 1000 ppm (1 mg/mL = 1000 μg/mL) in 80% MeOH. All samples were vortexed, sonicated, and centrifuged for 5 min at 14,000 rpm at 20 °C. All samples and standards were filtered through 0.22 μm CA filters prior to injection.

Samples were analyzed using an UHPLC–ESI–Orbitrap Exploris™ 120 mass spectrometer (Thermo Fisher Scientific, Waltham, MA, USA), and compounds were identified and quantified based on retention times, fragmentation patterns and external calibration curve compared to analytical standards (R^2^ values of calibration curves were over 0.99) [26]. Since the LC-HRMS method was employed for the comparative quantification of selected marker compounds across extracts obtained under different experimental conditions, method validation was limited to the evaluation of linearity and repeatability, which were considered appropriate for the purposes of the present study. Quantification was performed within the validated linear concentration range using external calibration curves of standards. UHPLC analyses were performed using a reverse-phase system (Vanquish UHPLC, Thermo Scientific, Waltham, MA, USA) equipped with a binary pump. Separation was achieved on a Phenomenex column (1.6 μm PS C18, 100 Å, 100 × 2.1 mm). The mobile phases consisted of water with 0.1% formic acid (A) and methanol with 0.1% formic acid (B), using the following gradient program: 0–5 min, 5–35% B; 5–25 min, 35–100% B; and 25–35 min, 100% B. Prior to analysis, both mobile phases were filtered through a 0.22 μm membrane. The flow rate was set at 0.2 mL/min, with a total run time of 35 min, an injection volume of 5 μL, and a column temperature maintained at 30 °C.

Mass spectrometric detection was carried out in both positive and negative ionization modes, applying a spray voltage of 3500 V. Full MS and data-dependent MS/MS (dd-MS^2^) scans were acquired at a resolution of 120,000 over a *m*/*z* range of 80–1200. Nitrogen was used as the collision gas, and fragmentation was achieved by collision-induced dissociation (CID) at 30 eV. The ion transfer tube and vaporizer temperatures were set at 300 °C and 280 °C, respectively. Sheath gas, auxiliary gas, and sweep gas flow rates were adjusted to 40, 20, and 0, respectively. Internal mass calibration was performed using the EASY-IC™ system (Thermo Fisher Scientific, Waltham, MA, USA).

Quantification was carried out using calibration curves of reference standards, and data processing was performed with Chromeleon 7.3.1 software (Thermo Scientific™ Dionex™).

The phytochemical profile of the optimized olive leaf extract (OE) was characterized using the same analytical method reported above.

### 2.6. Preparation of Liposomal Formulations

Liposomes were prepared by dispersing the soy phosphatidylcholine (purity > 94%) P90G, a cationic lipid (DOTAP) and the optimized *O. europaea* extract (OE) in phosphate-buffered saline (PBS) at pH 7.4 in the presence of hydrophilic surfactant polysorbate 80 (Tween80; HLB 14.9), according to the quantities reported in Table 2, and sonicating this dispersion with an ultrasonic disintegrator (Soniprep 150 plus; MSE Crowley, London, UK) [26].

The sonication setup consisted of a sequence of 7 cycles of 5 s and 1 cycle of 3 s active bursts, each cycle followed by 2 s pauses to prevent excessive heating. Preliminary optimization studies involving the calibration of both duration and intensity of sonication indicated that alternative sonication setups resulted in the formation of large (>150 nm) and polydispersed (polydispersity index ≫ 0.3) uncoated liposomes.

Subsequently, liposomes were coated with 0.5% (*w*/*v*) Eudragit^®^ L100 in PBS at a 1:1 (*v*/*v*) ratio, maintaining the dispersion under magnetic stirring for 20 min at room temperature to ensure homogeneous distribution of the polymer on the vesicle surface.

Empty uncoated and Eudragit-coated liposomes were prepared for proper comparisons.

### 2.7. Characterization of Liposomal Formulations

The Mean Diameter (MD), Zeta Potential (ZP), and Polydispersity Index (PI) of all liposome formulations were determined using a Zetasizer nano-ZS (Malvern Panalytical, Worcestershire, UK) via dynamic and electrophoretic light scattering at 25 °C. Results are reported as means of *n* > 10 replicates [26].

The liposome formulations were imaged via cryogenic-Transmission Electron Microscopy (cryo-TEM). A 3.5 µL aliquot of the samples were applied onto a glow-discharged 200-mesh Quantifoil (Großlöbichau, Germany) R1.2/1.3 holey copper grid. The grid was blotted for 6 s and vitrified by plunge-freezing into liquid ethane using a Vitrobot Mark IV (FEI Company/ThermoFisher Scientific, Waltham, MA, USA) operated at 4 °C and approximately 100% relative humidity. Grids were screened using a Talos Arctica cryo-electron microscope (FEI Company/ThermoFisher Scientific) operated at 200 kV.

Micrographs were acquired at a nominal magnification of 79,000× using a Falcon 4i direct electron detector equipped with a Selectris energy filter. The energy filter slit width was set to 10 eV. Images were recorded using SerialEM with an exposure time of 4 s, a total electron dose of 30 e^−^ Å^−2^, and a defocus range of −2.0 to −4.0 µm.

The liposomal formulations were dialyzed in 2 L of water to remove the extracts’ compounds not incorporated into the vesicles. After 2 h of gentle stirring, both the non-dialyzed and dialyzed liposomal dispersions were diluted (1:50 *v*/*v*) with methanol to disrupt the vesicles and allow quantification by LC-MS/MS (see Section 2.5) of the extract’s marker compounds, oleuropein and hydroxytyrosol. The entrapment efficiency (EE) was then calculated as the percentage ratio of the amount of each marker in the dialyzed formulations to the amount initially present in the non-dialyzed dispersions [23] according to Formula (3):

(3) $$Entrapment\;efficiency=\left(\frac{amount\;of\;oleuropein\;or\;hydroxytyrosol\;in\;dialyzed\;vesicles}{amount\;of\;oleuropein\;or\;hydroxytyrosol\;in\;non\text{-}dialized\;vesicles}\right)\times 100$$

### 2.8. Stability of Liposomal Formulations over Time and in Simulated Gastrointestinal Fluids

The long-term stability of the liposomal formulations, both uncoated and coated with Eudragit^®^ L100, and containing or not the OE, was assessed by macroscopic observation and periodic monitoring of key parameters such as MD, PI, and ZP, as described in Section 2.7. Measurements were performed at regular intervals for up to 5 months of storage.

Furthermore, stability under simulated gastrointestinal conditions was assessed by incubating liposomes containing the OE, uncoated or coated with Eudragit^®^, in simulated gastrointestinal fluids.

Liposomal dispersions were diluted 1:50 (*v*/*v*) with United States Pharmacopoeia simulated gastric fluid with pepsin from porcine stomach mucosa (USP SGF, pH 1.2) or with USP simulated intestinal fluid with pancreatin from porcine pancreas (USP SIF, pH 6.8) and incubated at 37 °C. The MD, PI, ZP were measured after 2 h of incubation in USP SGF (t_2h_) and 6 h of incubation in USP SIF (t_6h_) [26].

### 2.9. Cell Culture Conditions and Evaluation of Cell Viability

STC-1 cells, a murine, enteroendocrine cell line, were purchased from ATCC (Manassas, VA, USA) (CRL-3254^™^) and cultured in Dulbecco’s Modified Eagle Medium (DMEM) supplemented with 10% FBS, streptomycin (100 µg/mL), penicillin (100 units/mL), and glutamine (2 mM) and maintained in a humidified atmosphere (5% CO_2_ at 37 °C). Cell viability was tested in STC-1 cells using the MTT assay as previously reported [27]. STC-1 cells were treated with OE solution or OE Eudragit-coated liposomes at 0.1, 1, and 10 μg/mL for 2 h. The effects on cell viability were also investigated in STC-1 cells cultivated in 50 mM glucose to simulate a hyperglycemia condition induced 1 h before treatment. Empty Eudragit-coated liposomes were tested to exclude any potential effect induced by the nanocarriers. The dissolved formazan was quantified by measuring absorbance at 560 nm using a UV-Vis spectrophotometer (SPECTROstar^Nano^ BMG Labtech, Ortenberg, Germany). The effects of OE solution or liposomes in the hyperglycemia model are described below.

### 2.10. Intestinal Glucose Uptake

Glucose uptake was evaluated in STC-1 cells by using glucose 2-NBDG (100 µM), a fluorescent glucose derivative (λex 485 nm, λem 535 nm). Apigenin (50 µM) was used as positive control for the experiment. Fluorescence signals were measured using a Varioskan™ LUX multimode microplate reader (Thermo Fisher Scientific, Waltham, MA, USA), and the results were reported as the percentage of glucose uptake *vs.* untreated control cells [27].

### 2.11. Evaluation of Intracellular Reactive Oxygen Species (ROS)

Intracellular ROS were evaluated in STC-1 cells by adding 10 µM DCFH-DA and measuring fluorescence using a Varioskan™ LUX Multimode Microplate Reader (ThermoFisher Scientific, Waltham, MA, USA) (λex 485 nm and λem 515–540 nm). NAC (10 mM) was used as positive control of the experiment [23].

### 2.12. Glucagon-like Peptide 1 (GLP-1) Secretion

Active GLP-1 secretion was evaluated by GLP-1 ELISA kit (Invitrogen BMS2194, Carlsbad, CA, USA), following the manufacturer instructions [27].

### 2.13. Statistical Analysis

All data are presented as means ± standard deviation (SD). GraphPad Prism v. 8 Software Inc. (San Diego, CA, USA) was used to perform statistical analyses. Differences were considered statistically significant at *p* ≤ 0.05.

## 3. Results

### 3.1. Evaluation of RSM Adequacy

Once collected, the *O. europaea* leaves were air dried, resulting in a moisture loss of approximately 16.61%, corresponding to a reduction in weight from an initial value of 409.3 g for the fresh leaves to a final value of 341.3 g for the dried leaves. The dried leaves were then subjected to extraction.

Using the RSM approach, a Box–Behnken Design (BBD) matrix was constructed (Table 3) to evaluate the influence of solvent composition (0, 40, and 80% EtOH/H_2_O; factor A), temperature (25, 52.5, and 80 °C; factor B), and time (30, 135, and 240 min; factor C) on the extract’s antioxidant activity as well as on the extraction of phenolic compounds and characteristic specialized molecules of *O. europaea* leaves (tyrosol, hydroxytyrosol, and oleuropein).

The extraction yield obtained for all 17 experimental runs, together with that of the experimentally validated optimized extract, is reported in Supplementary Table S1. Extraction yields ranged from 5.15 to 42.90%, depending on the extraction conditions, while the optimized extraction afforded a yield of 37.95%.

ANOVA was performed to assess the effects of the studied variables, their interactions, and the overall statistical significance of the model (Table 4). In all cases, the data were analyzed without any transformations such as Natural Log, Base 10 Log, or Square Root functions. The statistical significance of the quadratic model was determined using the F-value and *p*-value. As shown in Table 4, F-values greater than 0 and *p*-values less than 0.05 were obtained for all response variables, demonstrating the adequacy of the quadratic model and thus that the selected polynomial equations adequately described the experimental data. This was further supported by the non-significant Lack of Fit (*p*-value > 0.05), confirming that the proposed model appropriately describes the experimental data.

The quadratic model reliability was evaluated using the coefficient of determination (R^2^), coefficient of variance (CV%), and adequate precision (Table 5). The models had high R^2^ values (0.96, 0.98, 0.89, 0.89, 0.92, and 0.98 for TPC, FRAP, DPPH, hydroxytyrosol, tyrosol, and oleuropein, respectively), with CV% always <20%, indicating good predictive power and experimental precision. Furthermore, adequate precision was always greater than 8 (up to >22 for oleuropein), confirming a good signal-to-noise ratio and the robustness of the model for exploring the experimental space (Table 5).

### 3.2. Impact of Extraction Variables on Total Phenolics and Antioxidant Properties

Second-order polynomial equations were used for each response to identify the effect of the independent variables on *O. europaea* leaf extracts’ antioxidant activity, total phenolic content, and characteristic specialized metabolites hydroxytyrosol, tyrosol, and oleuropein (Table 6).

For TPC, all three factors play a decisive role in linear terms, with positive effects of %EtOH, temperature, and time, but with a strong curvature associated mainly with the quadratic term of %EtOH (coeff. = −29.3507; *p*-value = 0.0050). This indicates that increasing the ethanol percentage promotes polyphenol extraction up to an optimal range, beyond which the yield tends to decrease, likely due to an excessive reduction in solvent polarity. Temperature also significantly increases total phenolic content, while time, although significant, has a less pronounced effect than the other two factors. A similar picture emerged for FRAP, which is strongly influenced by the three factors, but in this case, the response is dominated by the quadratic terms relating to %EtOH and temperature. The presence of large and highly significant quadratic coefficients of %EtOH (A^2^ coeff. = −154.3820; *p*-value = <0.0001) suggests a response surface with a well-defined maximum, in which the simultaneous optimization of solvent polarity and temperature allows the reducing power of the extract to be maximized. The interaction between temperature and time is also significant, which indicates that the effect of prolonged extraction on antioxidant power is not constant and depends strictly on the set temperature. The DPPH response shows a slightly different behavior, as the predominant factor is %EtOH, with a positive and statistically significant linear effect, while temperature and time are not as influential at the levels considered. However, the significance of the quadratic terms for %EtOH and temperature highlights a marked curvature on the surface, suggesting that the scavenger capacity does not increase indefinitely but reaches a maximum at intermediate conditions of solvent composition and temperature. The absence of significant interactions indicates that, for this response, the effects of the individual factors are mainly additive rather than synergistic.

**Figure 1:**
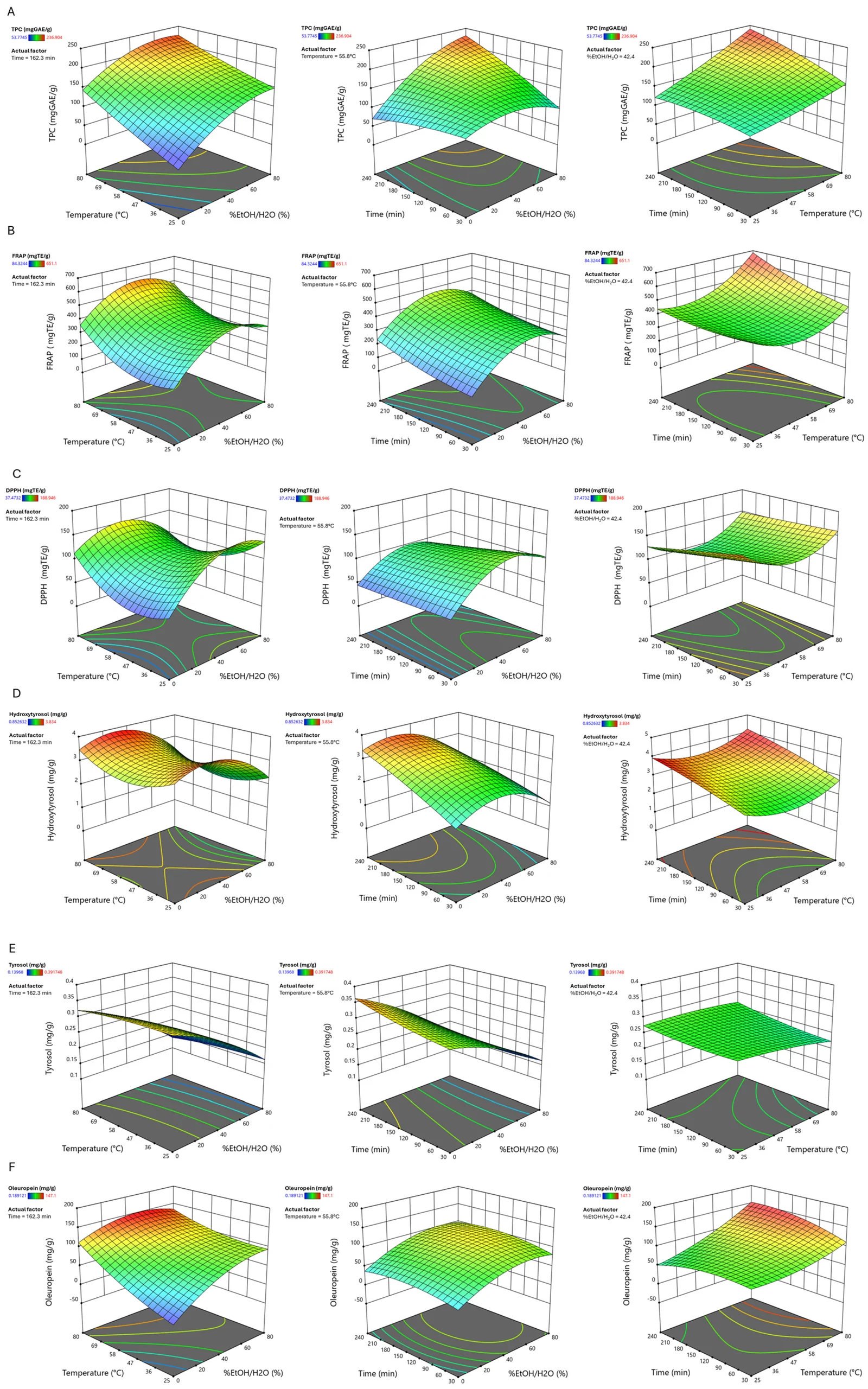
Three-dimensional surface plots illustrating the relationships between the independent variables (time, solvent, and temperature) and dependent variables: TPC (**A**), FRAP (**B**), DPPH (**C**), hydroxytyrosol (**D**), tyrosol (**E**), and oleuropein (**F**).

With regard to individual compounds, hydroxytyrosol shows a strong dependence on time and solvent composition: time has a positive and significant effect, suggesting that prolonging extraction favors the release of this more polar molecule, while %EtOH exerts an overall negative effect on a linear basis. This indicates that a high percentage of ethanol could penalize the extraction of hydroxytyrosol. However, the presence of a significant quadratic term for %EtOH suggests the existence of an optimum at intermediate polarity values. Tyrosol, on the other hand, is influenced almost exclusively by %EtOH, with a negative linear effect and no significant quadratic or interaction contributions, indicating a simpler response, essentially regulated by the polarity of the solvent. Finally, oleuropein represents the response for which the three factors, as quadratic forms, show the clearest and most articulated influence. %EtOH and temperature have positive and highly significant linear effects, confirming that the combination of a less polar solvent and higher temperatures is favorable for the solubilization of this secoiridoid, while time appears to be less decisive. However, the significance and negative sign of the quadratic terms of all factors, together with the presence of relevant interactions (particularly between %EtOH and temperature), reveal a response surface with a rather narrow optimum, outside of which the oleuropein yield tends to decrease. Figure 1 presents the surface plots of the interaction between dependent and independent variables.

Optimized parameters for each response are reported in Table 7.

### 3.3. Multiple Optimization Processes

The comparison between the predicted and experimentally obtained responses (Table 8) showed a generally good agreement, although some responses exhibited larger deviations than others. Such differences may reasonably reflect the intrinsic biological variability of plant-derived matrices and the multicomponent nature of the extract. Nevertheless, the experimental validation supported the ability of the model to identify extraction conditions leading to the desired phytochemical profile and antioxidant activity.

The primary objective of the optimization process was to identify the optimal combination of extraction parameters capable of producing an olive leaf extract with maximal total phenolic content and antioxidant activity, while ensuring a high yield of key bioactive compounds, including hydroxytyrosol, tyrosol, and oleuropein. Hence, based on the results obtained, the optimized extraction conditions were as follows: 30% EtOH/H_2_O, 80 °C, 240 min (Desirability 0.861) (Table 8).

These conditions featuring high temperature and prolonged extraction time, combined with an intermediate percentage of ethanol, represent a strategic compromise for maximizing both phenolic recovery and antioxidant performance. Hence, these extraction conditions predicted by the model were used to obtain the optimized extract. The optimized olive leaf extract (OE) exhibited a total phenolic content of 124.56 ± 9.57 mg GAE/g, accompanied by significant antioxidant activity, with FRAP and DPPH values of 472.92 ± 26.14 mg TE/g and 151.61 ± 2.77 mg TE/g, respectively. Quantitative analysis of key phenolic compounds revealed hydroxytyrosol at 2.238 ± 0.044 mg/g, tyrosol at 0.354 ± 0.028 mg/g, and oleuropein at 143.144 ± 4.914 mg/g. Although the experimental values were lower than those predicted by the model, they remained within the tolerance intervals, confirming the overall adequacy of the optimization approach.

### 3.4. Phytochemical Profile of Optimized Extract by LC-HRMS Analysis

In addition to the three characteristic compounds, hydroxytyrosol, tyrosol, and oleuropein, the phytochemical profile of the optimized extract (OE) displayed other 37 compounds, which were identified by comparing their retention times and MS/MS spectra with those of authentic standards, where available. The remaining compounds were identified by interpreting their MS and MS/MS spectra, along with information provided by the literature. Chromatograms of reference standards are provided in the Supplementary Materials.

**Figure 2:**
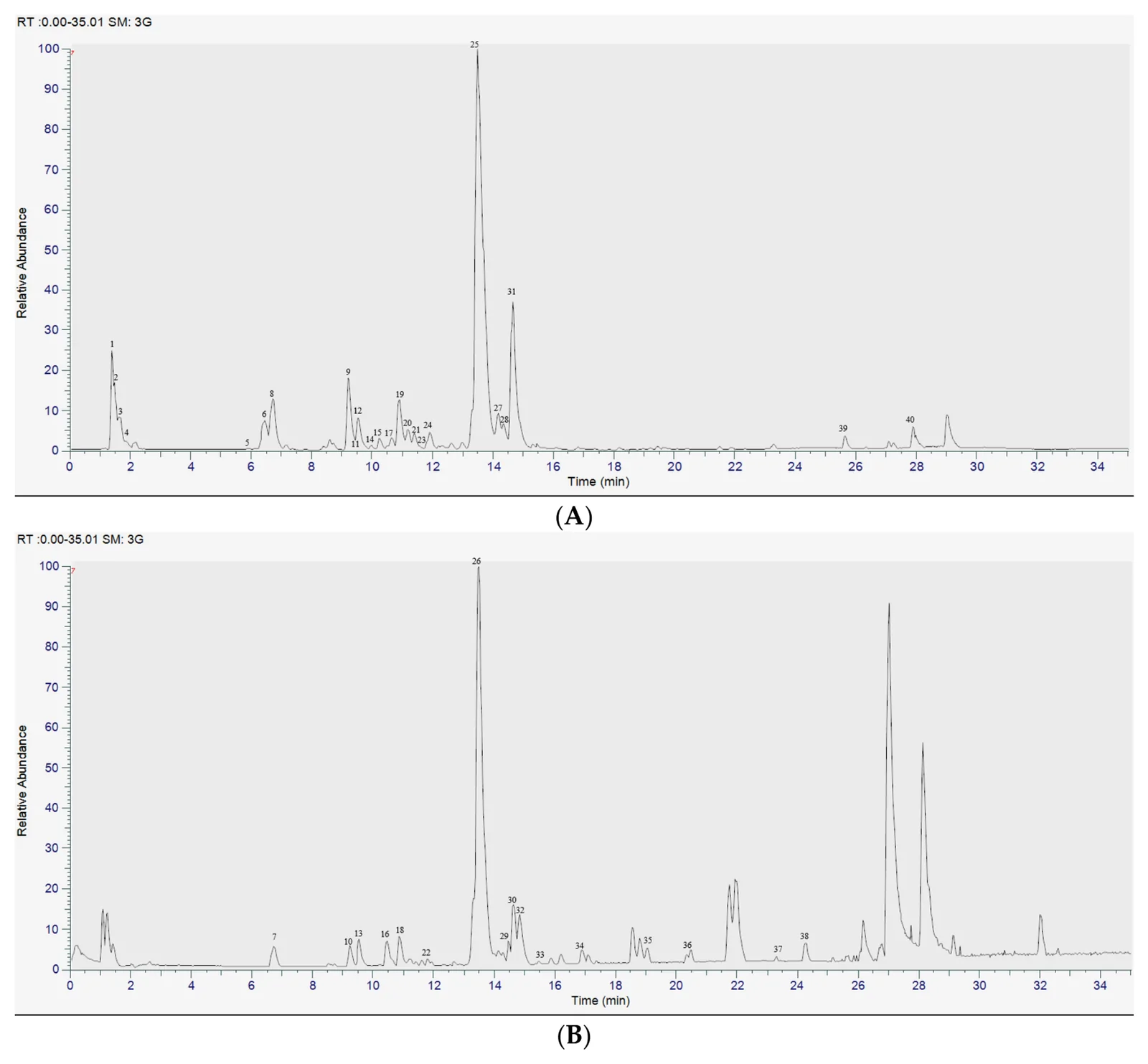
Base peak chromatogram for LC-HRMS analysis of optimized olive leaf extract in negative (**A**) and positive (**B**) modes.

The MS data of the identified compounds are reported in Figure 2 and Table 9, including *m*/*z* values associated with the proposed molecular formulas, mass errors, and principal MS/MS fragments, along with the tentative assignment of each peak. The obtained data reveal that the phenolic composition of OE included multiple classes, namely secoiridoids, phenols, flavonoids, benzoic acid derivatives, and triterpenes.

In the initial portion of the chromatogram (Figure 2; RT < 2 min), low-molecular-weight polar compounds were identified, including a polyol (*m*/*z* 181.0717), attributed to a sugar alcohol (**1**) [28], and the disaccharide gentiobiose (*m*/*z* 341.1087) (**2**) [29]. Quinic acid (**3**, 6.181 ± 0.104 mg/g) [30] and malic acid (**4**, 6.175 ± 0.104 mg/g) [31] were also quantified, both present in comparable concentrations and representative of the acidic organic fraction of the extract.

Among the simple phenols, the previously mentioned characteristic compounds described as one of the main components of olive leaves, 3-hydroxytyrosol (**5**, 2.328 ± 0.044 mg/g) [30,32,33] and tyrosol (**11**, 0.354 ± 0.028 mg/g) [34] were identified.

Peak **26** was attributed to dehydro-tyrosol (*m*/*z* 137.0596; formula C_8_H_8_O_2_). Fragmentation with ions at *m*/*z* 119 and a basic fragment at *m*/*z* 91 is compatible with a simple phenol structurally related to tyrosol. The presence of this compound suggests possible oxidative processes or metabolic transformations of the simple phenols present in the leaf matrix [35].

Furthermore, the glucoside of hydroxytyrosol (**6**) was particularly abundant (37.510 ± 0.417 mg/g) [36], indicating a significant presence of glycosylated forms.

Verbascoside (**23**, 2.144 ± 0.011 mg/g), a phenylpropanoid flavonoid known for its antioxidant properties, was also detected [30].

*O. europaea* is a source of secoiridoids that are primarily oleosides, which are typically esterified to a phenolic moiety in the Oleaceae group. Secoiridoids were indeed the most abundant fraction of the optimized extract. Oleuropein (**25**) was identified as the main compound in the extract, with a content of 143.144 ± 4.914 mg/g [30,32]. Several structurally related derivatives were also detected, including dimethyl-oleuropein (**21**, 3.605 ± 0.040 mg/g), hydroxyl-oleuropein (**20**, 1.373 ± 0.004 mg/g), and various oleuropein-type compounds (compound **27**, 10.918 ± 0.323 mg/g and compound **31**, 37.405 ± 0.008 mg/g), confirming the high biosynthetic activity of the secoiridoid pathway typical of *O. europaea* [30,32].

Oleoside-type compounds attributed to peaks **8**, **9**, and **12** [35] and derivatives of glycosylated elenolic acid (**15** and **19**) were also identified [30,35], further indicating the complexity of iridoid metabolism.

In the non-polar portion of the chromatogram, pentacyclic triterpenes such as maslinic acid (**39**, 1.392 ± 0.029 mg/g) [35] and ursolic acid (**40**, 1.089 ± 0.023 mg/g) [37], components frequently found in *O. europaea* matrices, were identified.

Several compounds were tentatively identified by comparison with literature data. For example, peak **14** at retention time of 9.98 min was attributed to globularitol (*m*/*z* 519.1721), a compound belonging to the class of glycosylated polyphenolic derivatives, with the predicted molecular formula C_22_H_32_O_14_. The MS/MS fragmentation pattern, characterized by ions at *m*/*z* 295 and 235 and the basic fragment at *m*/*z* 193, is consistent with what was reported in the literature [31].

Peak **22** (RT = 11.60 min) was assigned to an isomer of rutin (*m*/*z* 611.1607); in fact, the characteristic fragments at *m*/*z* 449 and 287 are compatible with the loss of the rutinosidic disaccharide moiety and the formation of the flavonol aglycone [35].

Peak **24** was identified as 2-(2-ethyl-3-hydroxy-6-propionylcyclohexyl) acetic acid glucoside (*m*/*z* 403.1972 and formula C_19_H_32_O_9_). The fragmentation pattern is consistent with the presence of a glycosylated terpenoid derivative. These compounds are related to the iridoid and secoiridoid biosynthetic pathways [28].

Peak **36** (RT = 20.36 min) was identified as *N*,*N*-dimethyl-safingol (*m*/*z* 330.3366), a compound belonging to the class of sphingolipids or long-chain amino derivatives, typically associated with the lipid fraction of the plant matrix. Its presence, detected in the nonpolar portion of the chromatogram, highlights the lipid component of the extract in addition to the predominant phenolic fraction [38].

Several peaks remained unidentified, despite presenting defined fragmentation patterns, suggesting the presence of additional specialized metabolites potentially characteristic of the extract that require further investigation.

### 3.5. Liposome Production and Characterization

The liposomal formulations were characterized by size, homogeneity, surface charge, and entrapment efficiency of key extract’s compounds oleuropein and hydroxytyrosol (Table 10). Tyrosol content in liposomal formulations was below the quantification limit of the analytical method used, making its quantitative determination not reliable in the formulations analyzed. Therefore, the entrapment efficiency of tyrosol is not reported.

The mean diameter of the empty liposomes was 74 ± 3.0 nm, with a polydispersity index of 0.27 ± 0.01, which indicates a homogeneous size distribution. Incorporation of the extract did not cause appreciable changes in either the mean diameter (73 ± 2.3 nm) or the PI (0.27 ± 0.01), suggesting that the extract components, while distributing between the aqueous core and the phospholipid bilayer according to their polarity/solubility characteristics, did not affect the structural organization of the vesicles.

Subsequent coating with Eudragit^®^ L100 resulted in a significant increase in size, with values of 105 ± 10.1 nm for empty Eu-liposomes and 101 ± 6.1 nm for extract-containing Eu-liposomes, accompanied by a decrease in homogeneity (PI ≥ 0.46). This behavior is consistent with the formation of a surface polymer layer around the vesicles induced by electrostatic interactions. Indeed, uncoated liposomes had a positive surface charge (approx. +8.0 ± 2.1 mV) due to the presence of the cationic lipid DOTAP that could interact with the anionic polymer Eudragit^®^. The reversed zeta potential detected for the Eu-liposomes (−17 ± 3.5 mV) confirmed this electrostatic interaction and the deposition of the polymer on the liposomes’ surface.

The entrapment efficiency (EE%) of both the uncoated and Eu-liposomes was determined by the dialysis method, a widely used approach for the characterization of polyphenol-loaded liposomal formulations. In particular, EE % was calculated based on the encapsulated amounts of oleuropein and hydroxytyrosol. High entrapment efficiencies were obtained, exceeding 60% for oleuropein and 95% for hydroxytyrosol.

**Figure 3:**
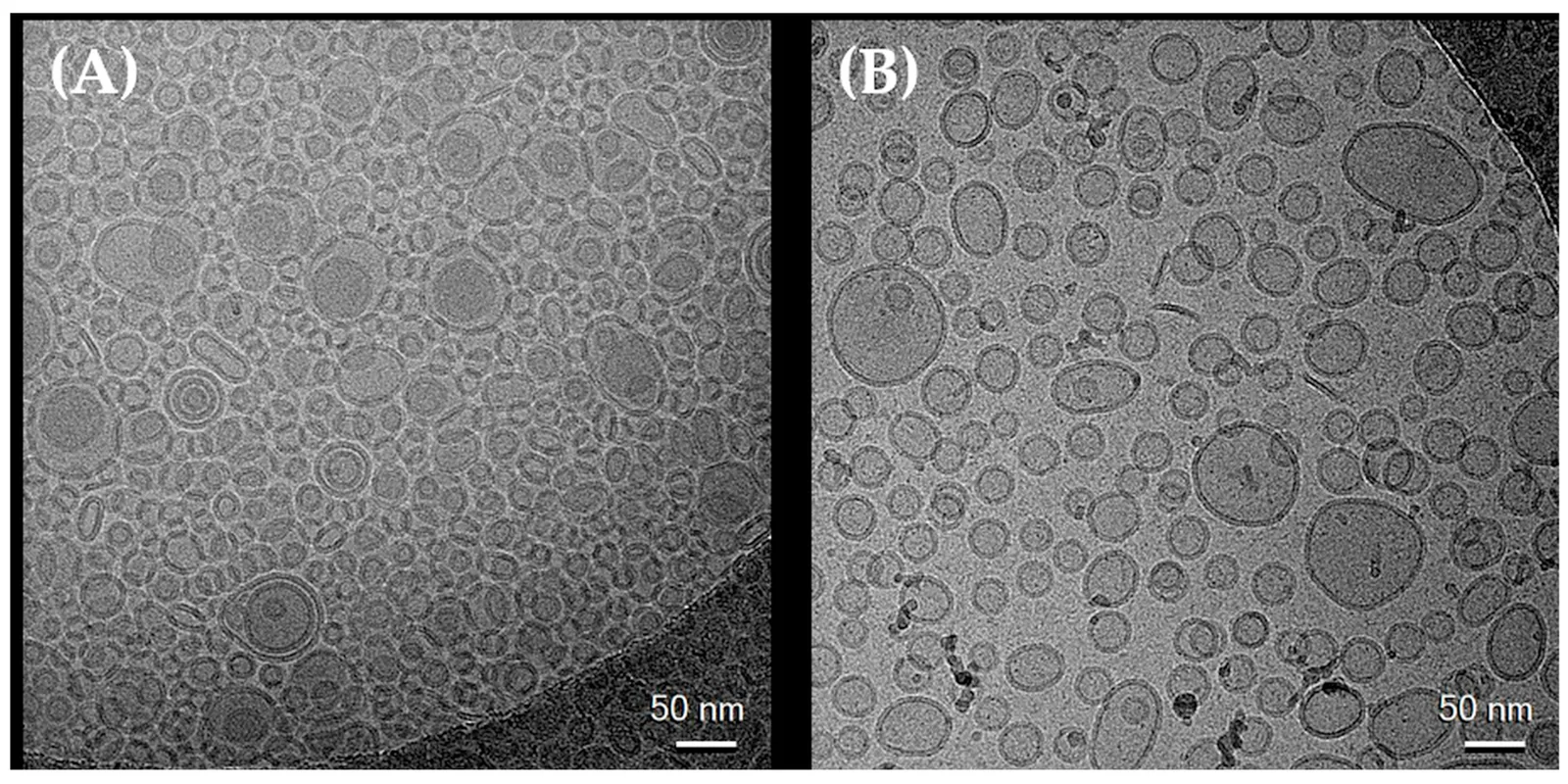
Cryo-TEM micrographs of optimized olive leaf extract uncoated liposomes (**A**) *vs.* Eu-liposomes (**B**).

A morphological assessment of the extract-containing uncoated liposomes and Eu-liposomes was performed by cryo-TEM. Representative micrographs are presented in Figure 3. OE uncoated liposomes were mainly unilamellar, spherical, and larger than 50 nm, although some bi-/tri-lamellar vesicles were also detected (Figure 3A). OE Eu-liposomes were spherical unilamellar vesicles that appeared larger, less densely packed, and less homogeneous than the uncoated liposomes (Figure 3B). The larger vesicle size and broader size distribution observed in OE Eu-liposomes can be reasonably ascribed to the arrangement of the Eudragit onto the liposome surface, which is often non-uniform across all vesicles. This morphological observation aligns with the values of average size and polydispersity index obtained from dynamic light scattering measurements (Table 10), demonstrating the convergence of the two techniques.

### 3.6. Storage Stability of Liposomal Formulations

All liposomal formulations were stored at 4 °C for 5 months and monitored at regular intervals by macroscopic observation and evaluation of key descriptive parameters including mean diameter, polydispersity index and zeta potential.

As shown in Table 11, the uncoated liposomes showed good stability over time. After 5 months of storage, a slight increase in polydispersity index (approx. 0.3 *vs.* 0.27) and zeta potential (≥+12 *vs.* +8 mV) was observed, while the mean diameter remained essentially unchanged (>70 nm).

Eu-liposomes exhibited a progressive reduction in mean diameter and polydispersity index (Table 11) during the monitored storage period, regardless of the presence of the extract. After 5 months, the mean diameter was set at around 92 nm and the polydispersity index at 0.38, compared to initial values at >100 nm and 0.46, respectively. These decreases can be attributed to a structural rearrangement of the polymer coating on the vesicle surface, with possible compaction of the Eudragit^®^ layer and consequent reduction in the initial dimensional heterogeneity. This phenomenon could reflect a progressive balance between the electrostatic interactions of the anionic polymer with the cationic surface of the liposomes and the inter-vesicle repulsion forces present in the colloidal system.

The zeta potential remained essentially unchanged over time (−15 mV; Table 11), suggesting that the negative surface charge induced by the polymer coating remained stable and that no significant polymer detachment or vesicle aggregation occurred.

Since the Eu-liposomes containing the optimized olive leaf extract were designed for oral administration, their stability was evaluated under conditions simulating the gastrointestinal environment. To ensure effective protection of the extract, the vesicles must maintain their structural integrity in a context characterized by wide pH variations and high ionic strength. To this end, the nanoformulations were incubated at 37 °C in SGF or SIF and the mean diameter, polydispersity, and zeta potential were measured immediately after 2 h or 6 h, respectively.

As reported in Table 12, after 2 h in SGF, uncoated liposomes showed a slight increase in mean diameter (83 ± 1.3 nm *vs.* 73 ± 2.3 nm), while the polydispersity index (0.28 ± 0.04) and zeta potential (+4 ± 2.2 mV) remained essentially unchanged from the initial values. A similar behavior was observed after 6 h in SIF, with the only difference being a marked shift in zeta potential toward negative values, attributable to the ionic composition of the medium (particularly the presence of anionic species such as HPO_4_^2−^), which modifies the surface layer.

Eu-liposomes maintained a nearly constant size (≈100 nm) in both SGF and SIF, with a low polydispersity index (≪0.46; see Table 10), which indicates a more homogeneous size distribution under stress conditions. No statistically significant difference (*p* = 0.072) was observed for the polydispersity index after incubation in SGF and SIF. The zeta potential was influenced by the ionic environment (−9 ± 5.2 mV in SGF and −25 ± 6.0 mV in SIF), reflecting the interactions between the anionic polymer coating and the ionic species present in the simulated fluids. Indeed, the difference in pH and salt concentrations between SGF and SIF alters the electrical double layer surrounding the polymer-coated vesicles, leading to different zeta potential values, thereby affecting the interactions between neighboring vesicles.

### 3.7. Eu-Liposomal Formulation Enhanced Extract’s Protective Effect Against ROS in Hyperglycemic Cells

Since this study aimed to evaluate the anti-hyperglycemic activity of the optimized olive leaf extract (OE), a high-glucose stress condition was simulated by culturing STC-1 intestinal cells in the presence of 50 mM glucose for 3 h. The biological activity of the OE was directly compared with that of its nanoformulation (OE Eu-liposomes).

**Figure 4:**
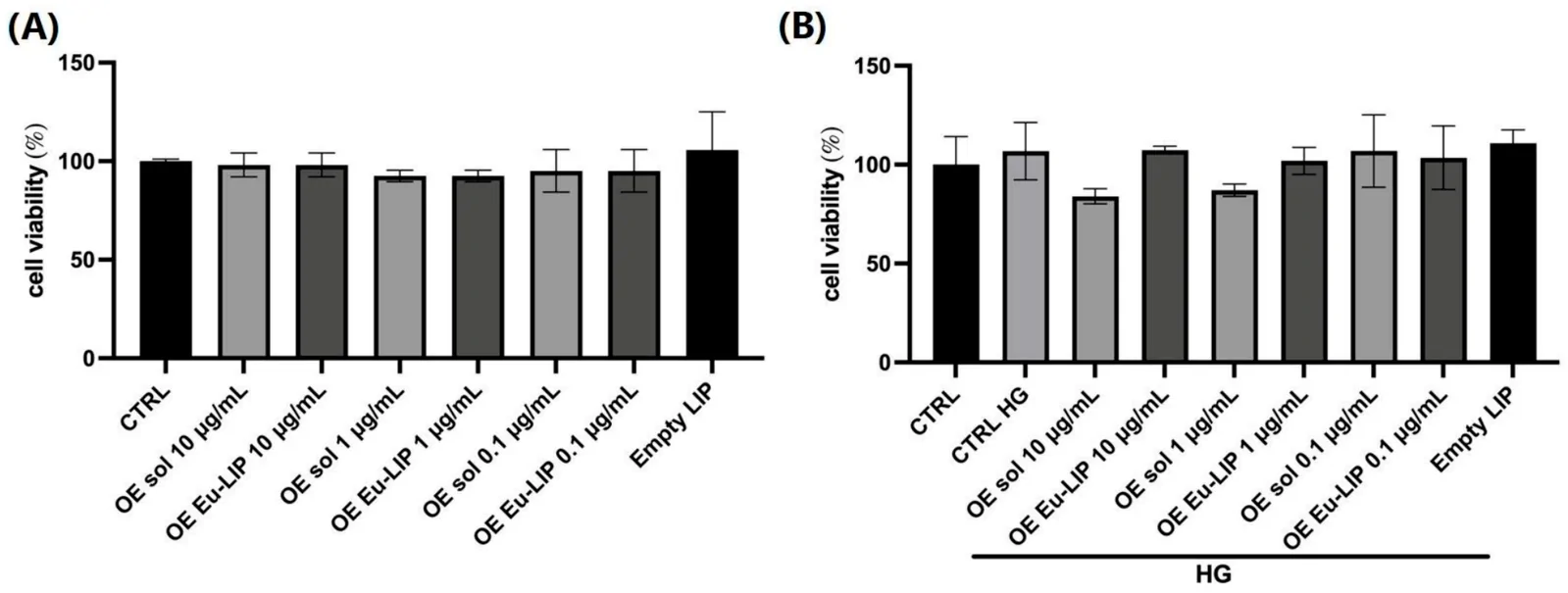
Assessment of cell viability using the MTT assay in STC-1 cells treated with optimized olive leaf extract in solution (OE sol) or in Eu-liposomes (OE Eu-LIP) at concentrations of 10, 1, and 0.1 μg/mL for 2 h under basal conditions (**A**) and under hyperglycemic (HG) stress conditions (**B**). No significant differences were observed compared to the control (CTRL), indicating the absence of cytotoxicity at the concentrations tested.

One hour after the induction of hyperglycemia, the cells were treated with OE in solution (in DMSO) and OE Eu-liposomes at different concentrations (0.1–10 µg/mL). Empty Eu-liposomes were included in the test to rule out a potential effect related to the components of the nanoformulation. First, the effect of the extract and the Eu-liposomes on the metabolic activity of the cells under basal conditions was evaluated by MTT assay. As shown in Figure 4A, neither the extract in solution nor the extract in the Eu-liposomes showed significant effects on cell metabolic activity. These results were also confirmed in the induced hyperglycemia model (Figure 4B), concluding that after 2 h, no concentration tested induced cytotoxic effects.

**Figure 5:**
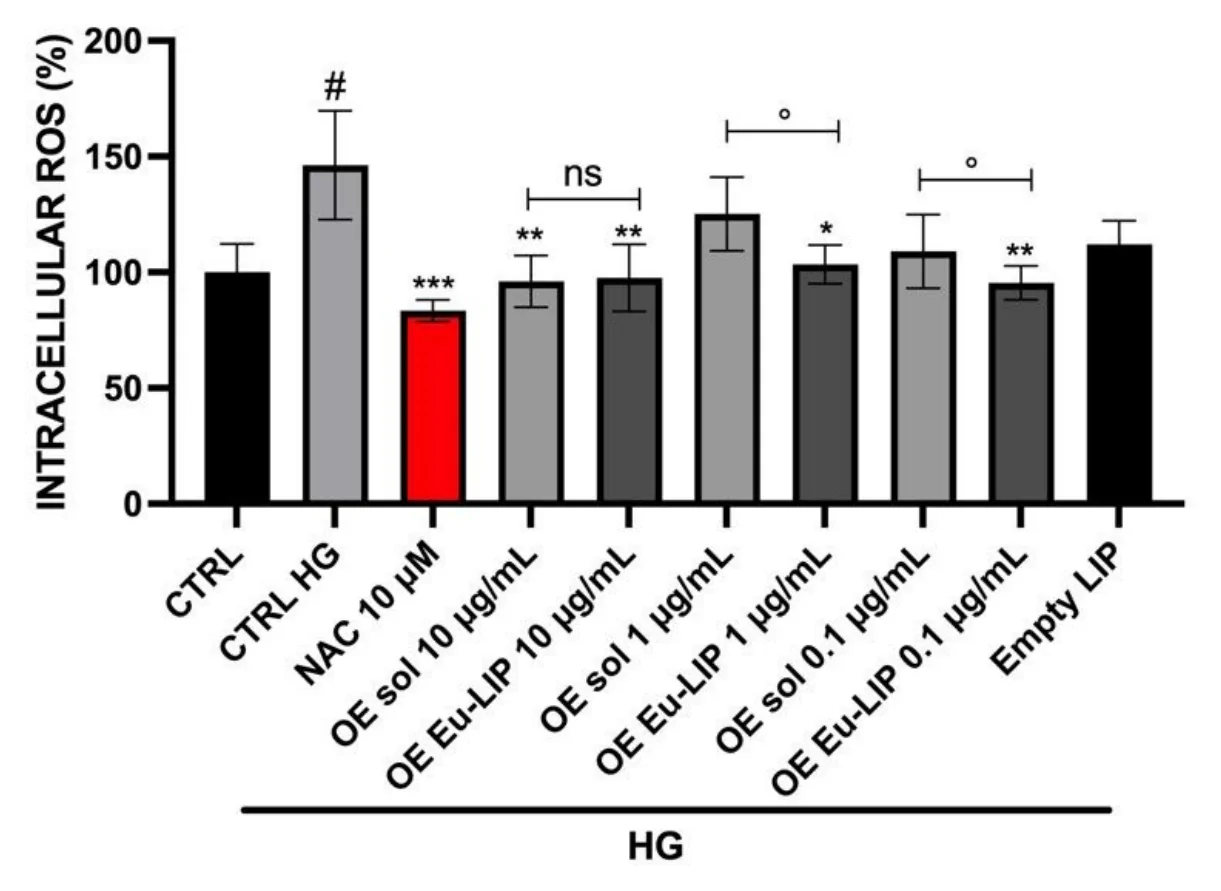
Effect of optimized olive leaf extract in solution (OE sol) or in Eu-liposomes (OE Eu-LIP) on intracellular ROS generation in STC-1 cells under high glucose conditions. Data are expressed as the mean ± SD of three independent experiments (*n* = 3) and were analyzed by one-way ANOVA followed by Tukey’s post hoc test. *** *p* < 0.001, ** *p* < 0.01, #/*/° *p* < 0.05. [° comparison between OE sol and OE Eu-LIP; # comparison between untreated hyperglycemic cells (CTRL HG) and untreated normoglycemic cells (CTRL); * comparison between HG treated cells and CTRL HG].

It has been widely demonstrated that hyperglycemia induces a marked increase in ROS production. For this reason, the protective effect of olive leaf extract, in solution and in Eu-liposomes, on oxidative stress induced by high glucose concentrations in STC-1 cells was evaluated using the DCFH-DA fluorescent probe. The probe, which converts intracellularly to DCF in the presence of ROS, generates a fluorescent signal proportional to ROS levels. The measured fluorescence showed that the induction of hyperglycemia (CTRL HG) causes a slight but statistically significant increase in ROS levels compared to cells grown in a normoglycemic environment (CTRL). However, treatment of cells with the liposomal extract and the extract in solution significantly reduced ROS production, restoring basal levels, at the highest concentration tested (10 µg/mL; Figure 5). Interestingly, the Eu-liposomes enhanced the effect of the extract, showing significant differences, especially at the lowest concentration tested (0.1 µg/mL; Figure 5). The empty Eu-liposomes did not induce significant differences, thus demonstrating that the formulation itself is inert.

### 3.8. Free and Liposomal Extract Reduce Glucose Uptake and GLP-1 Secretion in Hyperglycemic Cells

STC-1 cells are widely employed as an *in vitro* model to screen compounds capable of modulating gastrointestinal hormone secretion, including GLP-1. This hormone plays a key role in glucose homeostasis by promoting insulin secretion and suppressing glucagon release.

**Figure 6:**
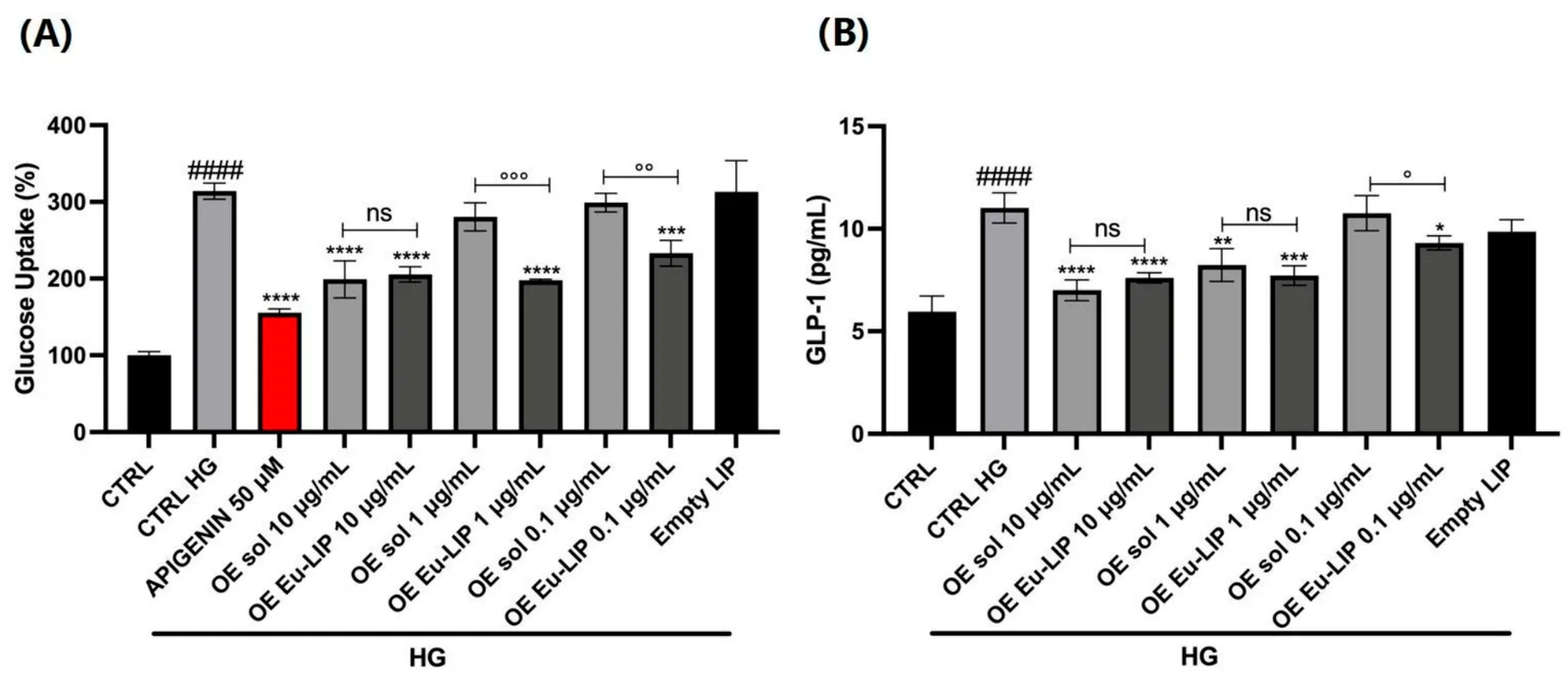
Effect of optimized olive leaf extract in solution (OE sol) or in Eu-liposomes (OE Eu-LIP) on intestinal glucose uptake (**A**) and GLP-1 secretion in STC-1 cells under high glucose conditions (**B**). Data are expressed as the mean ±SD of three independent experiments (n = 3) and were analyzed by one-way ANOVA followed by Tukey’s post hoc test. ####/**** *p* < 0.0001, ***/°°° *p* < 0.001 **/°° *p* < 0.01, */° *p* < 0.05. [° comparison between OE sol and OE Eu-LIP; # comparison between untreated hyperglycemic cells (CTRL HG) and untreated normoglycemic cells (CTRL); * comparison between HG treated cells and CTRL HG].

2-NBDG probe is a fluorescent glucose analogue widely used to evaluate cellular glucose transport. The 2-NBDG probe allowed us to directly and quantitatively monitor carbohydrate absorption at the cellular level. Our results confirmed that the experimental induction of a hyperglycemic environment caused a significant increase in intestinal glucose uptake, approximately three times higher than that observed under normoglycemic conditions, as shown in Figure 6A.

Treatment with OE sol showed significant inhibitory activity on glucose transport, particularly at the highest concentration tested (10 μg/mL). However, the use of the extract formulated in Eu-liposomes showed inhibitory effects on glucose uptake even at concentrations lower than that of the free extract. In addition, we investigated the ability of the same extract to stimulate GLP-1 secretion in STC-1 cells after 2 h of treatment with its solution or its nanoformulation at concentrations of 10, 1, and 0.1 μg/mL under hyperglycemic conditions. As shown in Figure 6B, a significant increase in GLP-1 secretion was evident in cells grown at high glucose (CTRL HG). The treatment with the OE, both in its free and liposomal form, resulted in a dose-dependent reduction in GLP-1 secretion, restoring the basal levels. As observed for the ROS levels, the liposomal formulation, particularly at lower concentrations, demonstrated a modestly greater inhibitory effect compared with the free extract.

## 4. Discussion

The results highlight the significant impact of extraction parameters on both the total phenolic content and antioxidant activity of *O. europaea* leaf extracts. Data analysis showed that ethanol concentration, temperature, and extraction time interactively influence the levels of phenolic compounds, particularly oleuropein, hydroxytyrosol, and tyrosol. In particular, it was demonstrated that the combination of %EtOH/H_2_O and temperature represents the primary driver of antioxidant activity (FRAP, DPPH) and efficacy of extracting phenolic compounds, while extraction time plays a secondary role. Solvent type, particularly for ethanol-containing mixtures, emerges as one of the most critical factors for achieving high-efficiency extraction. It has been widely demonstrated that ethanol mixed with water significantly improves the yield of phenolic compounds compared to using water alone due to a better interaction between solvents and target compounds [9]. The antioxidant activity assessed by FRAP was 472.92 ± 26.14 mg TE/g, which is higher than that reported by Lins et al. [39] for *O. europaea* leaf extract (281.8 ± 22.8 mg trolox equivalent/g dw). These data suggest a greater reducing power of our optimized olive leaf extract, potentially attributable, at least in part, to the different extraction method and the different mixture used [39]. Similarly, the total phenolic content evaluated using the TPC test yielded a value of 124.56 ± 9.57 mg GAE/g, which is higher than that reported by Mkaouar et al. [40] for the *O. europaea* leaf extract (66.63 ± 0.01). These data suggest a higher phenol content, possibly attributable to the different extraction temperature used (80 °C). Furthermore, the antioxidant activity of the extract was assessed using the FRAP test, yielding a value of 472.92 ± 26.14 mg TE/g, higher than that reported (30.1 ± 0.1 g Trolox/100 g DW) for *O. europaea* leaf extract in the study by Hayes et al. [1]. The observed increase could be attributed to the different extraction solvents used and the different extraction times.

The antioxidant activity of olive leaf extracts can be exploited to neutralize excess ROS within the human body. For example, hyperglycemia is known to induce ROS and can compromise endogenous antioxidant defense systems through various mechanisms. The introduction of antioxidants is considered a crucial strategy to reduce oxidative stress caused by hyperglycemia [41].

To investigate the biological activity of the optimized olive leaf extract in hyperglycemia, we used a cell model that involves high glucose exposure of STC-1 enteroendocrine cells, representing an *in vitro* system of acute glucotoxic stress. Intestinal glucose absorption is a key step in the regulation of postprandial blood glucose levels, especially in pathological conditions such as T2DM, where there is a marked alteration in intestinal glucose transporters [42]. It is known that a hyperglycemic environment can induce an increase in the expression and apical recruitment of GLUT2 in intestinal cells, thus increasing glucose absorption capacity and contributing to the exacerbation of postprandial blood glucose spikes. In line with what has been reported in the literature, our results confirm that the experimental induction of a hyperglycemic environment leads to a significant increase in intestinal glucose uptake.

The reduction in cellular glucose uptake observed following OE treatment is consistent with previous evidence attributing to oleuropein, one of the main phenolic compounds present in olive leaf extracts, the ability to inhibit intestinal glucose absorption, both through interference with digestive enzymes (such as sucrase) and through direct inhibition of intestinal transporters [43].

Optimization of the extraction process resulted in an extract characterized by higher *in vitro* antioxidant activity than that reported in the literature, as well as significant biological activity in STC-1 intestinal cells. The enrichment in secoiridoids and phenolic derivatives obtained via RSM optimization is particularly relevant. However, it is essential to consider the bioavailability and potential clinical applicability of the specialized metabolites responsible for these effects. The efficacy of orally administered plant extracts can be limited by phenomena such as gastrointestinal degradation, first-pass metabolism, uncontrolled distribution, and poor solubility in the physiological environment. Therefore, formulation strategies aimed at improving the stability and delivery of these metabolites may represent a promising approach. However, whether liposomal encapsulation improves their bioavailability remains to be established through dedicated permeability, pharmacokinetic and *in vivo* studies [26].

For these reasons, a liposome-based delivery strategy was proposed for the oral administration of the optimized olive leaf extract. Liposomes were coated with Eudragit^®^ L100, a gastro-resistant polymer that is insoluble in acidic environment and soluble at pH > 6. This polymer protects the vesicles in the stomach and promotes intestinal release of the active ingredients. Empty and uncoated liposomes were also prepared to evaluate the effect of the polymer coating and extract on the vesicles’ characteristics [26].

The formulations, subjected to various physico-chemical characterization tests, demonstrated good stability. Specifically, the collective data indicate that the Eu-liposomes achieved a stable colloidal configuration, without evidence of significant physical instability, making them suitable for the oral administration of the extract.

The results confirm that the combination of vesicle incorporation and polymer coating can provide protection to the extract in the gastrointestinal environment, a prerequisite for oral nanocarrier systems.

Eu-liposomes were used for in-cell evaluation, allowing us to assess whether technological optimization resulted in improved functional performance compared to the free extract. The nanoformulated extract showed inhibitory effects on glucose uptake even at concentrations lower than those of the extract in its free form. This greater efficacy could be explained by the improved bioavailability conferred by the liposomal formulation, which facilitates the stability and interaction of the phytocomplex with the cell membrane, promoting its intracellular release. Furthermore, the structure of the liposomes could allow for more prolonged interaction with intestinal cells, maximizing the inhibitory effect on glucose transport [44].

In addition to limiting glucose transport, the extract significantly decreased ROS levels in STC-1 cells exposed to high glucose. The protective effect of the optimized olive leaf extract is consistent with what has been reported in the literature, although not specifically associated with the cell model used in this study. For example, a study on oleuropein-enriched extract from olive leaves showed a dose-dependent reduction in ROS production in bovine epithelial cells (BME UV1 line) stimulated with hydrogen peroxide [45]. Similarly, in RAW 264.7 macrophages, olive leaf extract attenuated oxidative stress induced by free fatty acids (palmitate) by reducing intracellular ROS with increased expression of antioxidant genes, such as Nrf2, Hmox 1, and SOD2 protein [46]. Our results also suggest that the incorporation into liposomes not only maintains but also enhances the antioxidant effect of the extract. These observations are consistent with previous studies, albeit focused on single compounds. González-Ortega et al. [47] demonstrated that oleuropein encapsulated in phosphatidylcholine-based liposomes (PL-90 G) inhibited lipid peroxidation induced by oxidative stress, while being protected from degradation under UV exposure and in acidic environment.

In addition to the action of the optimized olive leaf extract on ROS levels and intestinal glucose uptake, we investigated the ability of the extract to stimulate GLP-1 secretion. GLP-1 is secreted by intestinal L cells in response to nutrients, stimulates insulin, suppresses glucagon secretion, and modulates the feeling of satiety. High-glucose environments are known to promote GLP-1 secretion [48].

Interestingly, the optimized leaf extract reversed this stimulatory effect, lowering GLP-1 secretion despite continued high-glucose exposure. This finding may appear contradictory. However, the concurrent reduction in glucose uptake suggests that the extract may interfere with glucose transporter activity, limiting intracellular glucose sensing and downstream GLP-1 release [49]. Studies in human L-cell models (e.g., NCI-H716) have shown that reduced glucose uptake, whether due to transporter inhibition or metabolic overload, leads to blunted GLP-1 secretion, even in the presence of high extracellular glucose [50]. The treatment with the optimized leaf extract, both in its free and liposomal form, reduced GLP-1 secretion, restoring basal levels, indicating that the extract modulates GLP-1 secretion under the experimental conditions used. However, the biological significance of this finding remains to be clarified, since GLP-1 secretion is influenced by multiple nutrient-sensing pathways and the present *in vitro* model does not fully reproduce the physiological regulation of incretin release.

Taken together, these findings indicate that OE reduces cellular glucose uptake and oxidative stress under high-glucose conditions. The concomitant decrease in GLP-1 secretion may reflect changes in cellular glucose sensing, although additional studies are required to establish the underlying mechanisms. One possible explanation is that the reduced GLP-1 secretion reflects altered glucose sensing secondary to reduced glucose uptake; however, this hypothesis requires further experimental validation. Our finding also aligns with literature data, demonstrating that GLP-1 elevations often accompany oxidative stress stimuli, and that reduction in ROS correlates with decreased compensatory GLP-1 release [51].

Overall, the enhanced cellular responses observed with the liposomal formulation suggest that nanoencapsulation may improve the *in vitro* biological activity of olive leaf polyphenols. However, whether these findings translate into improved intestinal delivery or bioavailability remains to be determined. Moreover, we cannot exclude the possibility that the extract interferes directly with nutrient-sensing or secretory pathways, such as SGLT1 function, mitochondrial ATP production, or calcium signaling. Further experiments examining these pathways will be needed to confirm the specific mechanism of action.

Hence, the obtained results indicate that the optimized *O. europaea* leaf extract exhibits antioxidant activity and modulates glucose uptake and GLP-1 secretion under high-glucose conditions in STC-1 cells. These findings support further investigation of this extract as a promising functional ingredient, while additional studies in more physiologically relevant models and *in vivo* systems are required to establish its therapeutic potential and possible antidiabetic effects.

## 5. Conclusions

This work shows how integrating statistical extraction design, metabolomic profiling, and advanced nanotechnologies enables a coherent translational pathway from plant matrix to ready-to-deliver formulation.

In particular, extraction parameters, such as time, ethanol concentration, and temperature, have a decisive impact on the total phenolic content, antioxidant activity, and concentration of major bioactive compounds in *O. europaea* leaf extracts. In particular, the optimized extraction conditions of 30% *v*/*v* ethanol at 80 °C for 240 min allowed us to obtain an extract characterized by strong antioxidant activity and high concentrations of main phenolic compounds (hydroxytyrosol, tyrosol and oleuropein). The results confirm the high efficiency of the extraction protocol, supporting the use of the predictive model in the development of functional ingredients and pharmaceutical options.

Moreover, our results highlight that, in a hyperglycemic cell model, treatment with the optimized olive leaf extract reduces markers associated with cellular stress, such as glucose uptake and ROS production.

These findings suggest that the extract exerts a protective modulation of cell function, potentially reducing the need for compensatory GLP-1 secretion under hyperglycemic conditions. This insight may have implications for the development of functional agents aimed at modulating incretin hormone responses in the context of metabolic disorders. Furthermore, the liposomal formulation represents an improved delivery strategy for the extract, as it enhances its biological activity, allowing protective effects to be achieved at lower extract concentrations.

Future investigations will focus on complementary analytical approaches to better elucidate the distribution of polyphenols within the vesicles.

Additional studies will also address the quantitative bioavailability and intestinal transport mechanisms of the polyphenols using more complex *in vitro*, *ex vivo* and *in vivo* models to further validate the nutraceutical and pharmacological potential of the proposed nanoformulation. Moreover, expanded analytical validation, including determination of detection and quantification limits, will be considered to further strengthen the robustness of the analytical approach.

Finally, future work will explore formulation scalability, long-term storage under different environmental conditions, and the applicability of this delivery platform to other classes of plant-derived bioactives. These developments will provide deeper insight into the nanosystem, while the current results based on optimized extraction, physicochemical characterization, stability under simulated gastrointestinal conditions, and functional biological evaluation already show the effectiveness of the proposed liposomal formulation as a delivery platform for olive leaf polyphenols.

## Figures and Tables

**Table 1:** Actual and coded levels used in the design.

Independent Variables				Dependent Variables
**Levels**	**−1**	**0**	**1**	
**A-%EtOH/H_2_O**	0	40	80	^1^ DPPH mg TE/g ^2^ FRAP mg TE/g ^3^ TPC mg GAE/g Hydroxytyrosol mg/g Tyrosol mg/g Oleuropein mg/g
**B-Temperature °C**	25	52.5	80	
**C-Time min**	30	135	260	

^1^ DPPH: 2,2-Diphenyl-1-picrylhydrazyl expressed as milligrams of Trolox Equivalents per gram of dry extract (mg TE/g); ^2^ FRAP: Ferric Reducing Antioxidant Power expressed as milligrams of Trolox Equivalents per gram of dry extract (mg TE/g); ^3^ TPC: Total Phenolic Content expressed as milligrams of Gallic Acid Equivalents per gram of dry extract (mg GAE/g).

**Table 2:** Composition of the liposomal formulations.

	P90G	OE	DOTAP	Tween80	PBS	0.5% Eudragit^®^ in PBS
**Empty liposomes**	60 mg	-	5 mg	17 μL	983 μL	-
**OE** **liposomes**	60 mg	2 mg	5 mg	17 μL	983 μL	-
**Empty Eu-liposomes**	60 mg	-	5 mg	17 μL	983 μL	1000 μL
**OE Eu-liposomes**	60 mg	2 mg	5 mg	17 μL	983 μL	1000 μL

P90G, phosphatidylcholine; OE, optimized *O. europaea* extract; DOTAP, 1,2-Dioleoyloxy-3-trimethylammonium propane chloride; PBS, phosphate-buffered saline; Eu, Eudragit^®^ L100.

**Table 3:** Independent and dependent variables, with their respective code levels, and results for Total Phenolic Content (TPC), antioxidant activity (DPPH, FRAP), and the detected amounts of hydroxytyrosol, tyrosol, and oleuropein.

	Independent Variables			Dependent Variables					
Run	A Solvent (%EtOH/H_2_O)	B Temperature (°C)	C Time (min)	TPC ^1^ mg GAE/g	DPPH ^2^ mg TE/g	FRAP ^3^ mg TE/g	Hydroxytyrosol mg/g	Tyrosol mg/g	Oleuropein mg/g
1	40	25	30	121.803 ± 5.076 ^g,h^	188.946 ± 14.341 ^a^	458.843 ± 48.080 ^b,c^	3.197 ± 0.053 ^c,d^	0.295 ± 0.003 ^c,d^	81.437 ± 0.947 ^i^
2	80	52.5	30	89.002 ± 4.490 ^i,j^	91.901 ± 3.485 ^f^	250.452 ± 19.953 ^f,g^	0.853 ± 0.053 ^i^	0.140 ± 0.000 ^l^	74.344 ± 0.970 ^j^
3	0	25	135	53.774 ± 5.016 ^k^	37.473 ± 2.439 ^h^	136.211 ± 7.046 ^h,i^	3.043 ± 0.048 ^d^	0.307 ± 0.007 ^b,c^	0.189 ± 0.015 ^n^
4	0	52.5	240	70.513 ± 8.417 ^j,k^	55.053 ± 3.452 ^g,h^	222.164 ± 13.168 ^g,h^	3.619 ± 0.031 ^b^	0.392 ± 0.007 ^a^	36.440 ± 0.044 ^l^
5	80	25	135	138.163 ± 4.533 ^f,g^	141.653 ± 9.629 ^b,c^	325.225 ± 24.226 ^e,f^	2.107 ± 0.075 ^g^	0.174 ± 0.001 ^j^	101.293 ± 0.816 ^e,f^
6	40	52.5	135	159.286 ± 9.665 ^d,e,f^	136.616 ± 11.146 ^b,c,d^	381.351 ± 16.001 ^b,c,d,e^	2.787 ± 0.044 ^e^	0.269 ± 0.003 ^f,g^	102.350 ± 0.182 ^e,f^
7	0	52.5	30	94.461 ± 7.766 ^h,i,j^	49.691 ± 4.508 ^g,h^	84.324 ± 8.084 ^i^	1.278 ± 0.015 ^h^	0.313 ± 0.004 ^b^	22.701 ± 0.060 ^m^
8	40	80	240	227.703 ± 1.137 ^a,b^	130.656 ± 4.885 ^c,d^	651.123 ± 47.786 ^a^	3.834 ± 0.041 ^a^	0.250 ± 0.004 ^h^	127.192 ± 0.278 ^a^
9	40	52.5	135	140.895 ± 9.453 ^e,f,g^	98.381 ± 3.796 ^f^	367.468 ± 27.853 ^c,d,e^	2.757 ± 0.028 ^e^	0.231 ± 0.005 ^i^	85.820 ± 0.423 ^h^
10	80	80	135	198.190 ± 17.258 ^b,c^	124.401 ± 9.953 ^c,d,e^	469.148 ± 40.106 ^b^	2.423 ± 0.101 ^f^	0.163 ± 0.001 ^j,k^	130.287 ± 0.813 ^b^
11	40	52.5	135	170.499 ±13.354 ^c,d,e^	105.687 ± 4.608 ^e,f^	331.565 ± 19.642 ^e,f^	2.682 ± 0.047 ^e^	0.289 ± 0.001 ^d,e^	101.005 ± 0.718 ^f^
12	40	80	30	172.078 ± 16.267 ^c,d^	155.639 ± 6.058 ^b^	437.989 ± 36.463 ^b,c,d^	3.031 ± 0.064 ^d^	0.231 ± 0.001 ^i^	118.138 ± 0.795 ^c^
13	80	52.5	240	236.904 ± 14.914 ^a^	60.910 ± 3.722 ^g^	374.700 ± 34.501 ^b,c,e^	2.354 ± 0.069 ^f^	0.155 ± 0.002 ^k^	98.728 ± 1.178 ^g^
14	40	52.5	135	163.611 ± 10.687 ^d,e,f^	107.845 ± 4.262 ^e,f^	382.059 ± 29.432 ^b,c,e^	3.084 ± 0.034 ^d^	0.259 ± 0.002 ^g,h^	103.215 ± 0.493 ^e^
15	40	52.5	135	161.380 ± 8.142 ^d,e,f^	105.468 ± 4.246 ^e,f^	350.275 ± 31.584 ^d,e^	3.319 ± 0.065 ^c^	0.269 ± 0.008 ^f,g^	98.067 ± 0.956 ^g^
16	40	25	240	103.366 ± 3.448 ^h,i^	130.824 ± 11.212 ^c,d^	449.893 ± 41.499 ^b,c^	3.753 ± 0.075 ^a,b^	0.279 ± 0.006 ^e,f^	41.006 ± 0.116 ^k^
17	0	80	135	145.683 ± 10.626 ^d,e,f,g^	114.428 ± 9.637 ^d,e,f^	350.718 ± 38.381 ^d,e^	3.330 ± 0.058 ^c^	0.304 ± 0.006 ^b,c^	105.488 ± 0.587 ^d^

^1^ TPC: Total Phenolic Content expressed as milligrams of Gallic Acid Equivalents per gram of dry extract (mg GAE/g); ^2^ DPPH: 2,2-Diphenyl-1-picrylhydrazyl expressed as milligrams of Trolox Equivalents per gram of dry extract (mg TE/g); ^3^ FRAP: Ferric Reducing Antioxidant Power expressed as milligrams of Trolox Equivalents per gram of dry extract (mg TE/g). Results are expressed as mean ± standard deviation of mg/g dry extract. Different letters in each column indicate statistically significant differences (*p* < 0.05).

**Table 4:** ANOVA statistical analysis.

**Response 1: ^1^ TPC**						
**Source**	**Sum of Squares**	**df**	**Mean Square**	**F-Value**	***p*** **-Value**	
**Model**	40,632.52	9	4514.72	20.2	0.0003	significant
**A-%EtOH/H_2_O**	11,088.54	1	11,088.54	49.62	0.0002 *	
**B-Temperature**	13,329.15	1	13,329.15	59.65	0.0001 *	
**C-Time**	3245.41	1	3245.41	14.52	0.0066 *	
**AB**	254.11	1	254.11	1.14	0.3217	
**AC**	7384.39	1	7384.39	33.04	0.0007 *	
**BC**	1371.05	1	1371.05	6.14	0.0424 *	
**A^2^**	3627.21	1	3627.21	16.23	0.005 *	
**B^2^**	73.19	1	73.19	0.3275	0.585	
**C^2^**	210.3	1	210.3	0.9411	0.3643	
**Residual**	1564.3	7	223.47			
**Lack of Fit**	1077.36	3	359.12	2.95	0.1616	not significant
**Pure Error**	486.95	4	121.74			
**Cor Total**	42,196.82	16				
**Response 2: ^2^ FRAP**						
**Source**	**Sum of Squares**	**df**	**Mean Square**	**F-Value**	***p*** **-Value**	
**Model**	2.74 × 10^5^	9	30,472.79	34.04	<0.0001	significant
**A-%EtOH/H_2_O**	49,000.13	1	49,000.13	54.74	0.0001 *	
**B-Temperature**	36,292.62	1	36,292.62	40.54	0.0004 *	
**C-Time**	27,178.51	1	27,178.51	30.36	0.0009 *	
**AB**	1244.01	1	1244.01	1.39	0.277	
**AC**	46.18	1	46.18	0.0516	0.8268	
**BC**	12,323.04	1	12,323.04	13.77	0.0076 *	
**A^2^**	1.00 × 10^5^	1	1.00 × 10^5^	112.11	<0.0001 *	
**B^2^**	52,961.45	1	52,961.45	59.17	0.0001 *	
**C^2^**	2578.9	1	2578.9	2.88	0.1334	
**Residual**	6265.92	7	895.13			
**Lack of Fit**	4396.89	3	1465.63	3.14	0.1492	not significant
**Pure Error**	1869.03	4	467.26			
**Cor Total**	2.81 × 10^5^	16				
**Response 3: ^3^ DPPH**						
**Source**	**Sum of Squares**	**df**	**Mean Square**	**F-Value**	***p*** **-Value**	
**Model**	22,873.25	9	2541.47	6.04	0.0135	significant
**A-%EtOH/H_2_O**	3267.21	1	3267.21	7.77	0.027 *	
**B-Temperature**	85.98	1	85.98	0.2044	0.6649	
**C-Time**	1463.01	1	1463.01	3.48	0.1045	
**AB**	2218.71	1	2218.71	5.27	0.0553	
**AC**	340.4	1	340.4	0.8091	0.3983	
**BC**	274.54	1	274.54	0.6525	0.4458	
**A^2^**	9163.37	1	9163.37	21.78	0.0023 *	
**B^2^**	6851.95	1	6851.95	16.29	0.005 *	
**C^2^**	0.5971	1	0.5971	0.0014	0.971	
**Residual**	2945.13	7	420.73			
**Lack of Fit**	2061.15	3	687.05	3.11	0.1509	not significant
**Pure Error**	883.98	4	221			
**Cor Total**	25,818.38	16				
**Response 4: Hydroxytyrosol**						
**Source**	**Sum of Squares**	**df**	**Mean Square**	**F-Value**	***p*** **-Value**	
**Model**	9.37	9	1.04	6.51	0.0109	significant
**A-%EtOH/H_2_O**	1.56	1	1.56	9.75	0.0168 *	
**B-Temperature**	0.0336	1	0.0336	0.2099	0.6608	
**C-Time**	3.38	1	3.38	21.14	0.0025 *	
**AB**	0.0002	1	0.0002	0.0014	0.971	
**AC**	0.1762	1	0.1762	1.1	0.3289	
**BC**	0.0153	1	0.0153	0.0958	0.766	
**A^2^**	2.79	1	2.79	17.43	0.0042 *	
**B^2^**	1.59	1	1.59	9.92	0.0162 *	
**C^2^**	0.0311	1	0.0311	0.1942	0.6727	
**Residual**	1.12	7	0.16			
**Lack of Fit**	0.8331	3	0.2777	3.87	0.112	not significant
**Pure Error**	0.2869	4	0.0717			
**Cor Total**	10.49	16				
**Response 5: Tyrosol**						
**Source**	**Sum of Squares**	**df**	**Mean Square**	**F-Value**	***p*** **-Value**	
**Model**	0.0645	9	0.0072	8.5	0.005	significant
**A-%EtOH/H_2_O**	0.0584	1	0.0584	69.33	<0.0001 *	
**B-Temperature**	0.0014	1	0.0014	1.67	0.2372	
**C-Time**	0.0012	1	0.0012	1.42	0.2729	
**AB**	0	1	0	0.0211	0.8887	
**AC**	0.001	1	0.001	1.19	0.3118	
**BC**	0.0003	1	0.0003	0.3815	0.5563	
**A^2^**	0.0017	1	0.0017	2.04	0.1958	
**B^2^**	0.0002	1	0.0002	0.207	0.6629	
**C^2^**	0.0002	1	0.0002	0.2263	0.6488	
**Residual**	0.0059	7	0.0008			
**Lack of Fit**	0.0041	3	0.0014	3.07	0.1534	not significant
**Pure Error**	0.0018	4	0.0004			
**Cor Total**	0.0704	16				
**Response 6: Oleuropein**						
**Source**	**Sum of Squares**	**df**	**Mean Square**	**F-Value**	***p*** **-Value**	
**Model**	23,895.43	9	2655.05	34.6	<0.0001	significant
**A-%EtOH/H_2_O**	7188.45	1	7188.45	93.67	<0.0001 *	
**B-Temperature**	9599.75	1	9599.75	125.09	<0.0001 *	
**C-Time**	88.87	1	88.87	1.16	0.3175	
**AB**	1455.59	1	1455.59	18.97	0.0033 *	
**AC**	28.18	1	28.18	0.3672	0.5637	
**BC**	1202.57	1	1202.57	15.67	0.0055 *	
**A^2^**	2917.05	1	2917.05	38.01	0.0005 *	
**B^2^**	662.54	1	662.54	8.63	0.0218 *	
**C^2^**	793.1	1	793.1	10.33	0.0148 *	
**Residual**	537.19	7	76.74			
**Lack of Fit**	333.74	3	111.25	2.19	0.2321	not significant
**Pure Error**	203.46	4	50.86			
**Cor Total**	24,432.63	16				

^1^ TPC: Total Phenolic Content expressed as milligrams of Gallic Acid Equivalents per gram of dry extract (mg GAE/g); ^2^ FRAP: Ferric Reducing Antioxidant Power expressed as milligrams of Trolox Equivalents per gram of dry extract (mg TE/g); ^3^ DPPH: 2,2-Diphenyl-1-picrylhydrazyl expressed as milligrams of Trolox Equivalents per gram of dry extract (mg TE/g); df: Degree of freedom. F-values > 0 and * *p*-value < 0.05 were considered significant.

**Table 5:** Fit statistical analysis.

	^1^ TPC	^2^ FRAP	^3^ DPPH	Hydroxytyrosol	Tyrosol	Oleuropein
**Std. Dev.**	14.95	29.92	20.51	0.40	0.03	8.76
**Mean**	143.96	354.32	108.01	2.79	0.25	85.15
**^4^ C.V. %**	10.38	8.44	18.99	14.33	11.43	10.29
**R^2^**	0.96	0.98	0.89	0.89	0.92	0.98
**Adeq Precision**	16.38	25.61	8.27	10.06	10.22	22.56

^1^ TPC: Total Phenolic Content expressed as milligrams of Gallic Acid Equivalents per gram of dry extract (mg GAE/g); ^2^ FRAP: Ferric Reducing Antioxidant Power expressed as milligrams of Trolox Equivalents per gram of dry extract (mg TE/g); ^3^ DPPH: 2,2-Diphenyl-1-picrylhydrazyl expressed as milligrams of Trolox Equivalents per gram of dry extract (mg TE/g); ^4^ CV%: coefficient of variance; *p*-value < 0.05 was considered significant.

**Table 6:** Model regression equations.

	Intercept	A	B	C	AB	AC	BC	A^2^	B^2^	C^2^
**TPC ^1^**	159.134	37.2299	40.8184	20.1414	−7.9704	42.9663	18.5138	−29.3507	4.1692	−7.0673
***p*** **-values**		0.0002 *	0.0001 *	0.0066 *	0.3217	0.0007 *	0.0424 *	0.0050 *	0.5850	0.3643
**FRAP ^2^**	362.544	78.2625	67.3541	58.2865	−17.6353	−3.3980	55.5046	−154.3820	112.1530	24.7485
***p*** **-values**		0.0001 *	0.0004 *	0.0009 *	0.2770	0.8268	0.0076 *	<0.0001 *	0.0001 *	0.1334
**DPPH ^3^**	110.799	20.2089	3.2784	−13.5232	−23.5516	−9.2250	8.2846	−46.6508	40.3403	0.3766
***p*** **-values**		0.0270 *	0.6649	0.1045	0.0553	0.3983	0.4458	0.0023 *	0.0050 *	0.9710
**Hydroxytyrosol**	2.92569	−0.4416	0.0648	0.6502	0.0075	−0.2099	0.0619	−0.8139	0.6138	−0.0859
***p*** **-values**		0.0168 *	0.6608	0.0025 *	0.9710	0.3289	0.7660	0.0042 *	0.0162 *	0.6727
**Tyrosol**	0.26339	−0.0855	−0.01323	0.0122	−0.0021	−0.0158	0.0090	−0.0202	−0.0064	0.0067
***p*** **-values**		<0.0001 *	0.2372	0.2729	0.8887	0.3118	0.5563	0.1958	0.6629	0.6488
**Oleuropein**	98.0913	29.9759	34.6406	3.3330	−19.0761	2.6542	17.3391	−26.3211	12.5441	−13.7244
***p*** **-values**		<0.0001 *	<0.0001 *	0.3175	0.0033 *	0.5637	0.0055 *	0.0005 *	0.0218 *	0.0148 *

^1^ TPC: Total Phenolic Content expressed as milligrams of Gallic Acid Equivalents per gram of dry extract (mg GAE/g); ^2^ FRAP: Ferric Reducing Antioxidant Power expressed as milligrams of Trolox Equivalents per gram of dry extract (mg TE/g); ^3^ DPPH: 2,2-Diphenyl-1-picrylhydrazyl expressed as milligrams of Trolox Equivalents per gram of dry extract (mg TE/g); A: %EtOH/H_2_O, B: Temperature °C, C: Time min. * *p*-value < 0.05 was considered significant.

**Table 7:** Optimal conditions determined via numerical optimization of RSM models for each dependent variable.

	Solvent (%EtOH/H_2_O)	Temperature (°C)	Time (min.)	Predicted Value
**^1^ TPC**	78.304	63.298	239.04	242.534
**^2^ FRAP**	43.548	77.723	239.800	656.305
**^3^ DPPH**	62.758	25.000	30.000	185.090
**^4^ Hydroxytyrosol**	20.867	25.839	196.640	3.844
**^4^ Tyrosol**	0	47.661	240.000	0.364
**^4^ Oleuropein**	56.316	78.614	231.709	150.347

^1^ TPC: Total Phenolic Content expressed as milligrams of Gallic Acid Equivalents per gram of dry extract (mg GAE/g); ^2^ FRAP: Ferric Reducing Antioxidant Power expressed as milligrams of Trolox Equivalents per gram of dry extract (mg TE/g); ^3^ DPPH: 2,2-Diphenyl-1-picrylhydrazyl expressed as milligrams of Trolox Equivalents per gram of dry extract (mg TE/g); ^4^ Hydroxytyrosol, Tyrosol, and Oleuropein are expressed in mg/g.

**Table 8:** Optimized extraction conditions.

	Predicted Mean	Predicted Median	Std Dev	SE Mean	* 95% TI Low for 99% Pop	95% TI High for 99% Pop
**TPC ^1^**	217.267	217.267	14.949	13.127	123.196	311.338
**FRAP ^2^**	658.843	658.843	29.919	26.272	470.569	847.116
**DPPH ^3^**	149.949	149.949	20.512	18.012	20.872	279.026
**Hydroxytyrosol ^4^**	4.336	4.336	0.400	0.351	1.819	6.854
**Tyrosol ^4^**	0.295	0.295	0.029	0.025	0.112	0.477
**Oleuropein ^4^**	147.630	147.630	8.760	7.693	92.504	202.757

^1^ TPC: Total Phenolic Content expressed as milligrams of Gallic Acid Equivalents per gram of dry extract (mg GAE/g); ^2^ FRAP: Ferric Reducing Antioxidant Power expressed as milligrams of Trolox Equivalents per gram of dry extract (mg TE/g); ^3^ DPPH: 2,2-Diphenyl-1-picrylhydrazyl expressed as milligrams of Trolox Equivalents per gram of dry extract (mg TE/g); ^4^ Hydroxytyrosol, Tyrosol, Oleuropein are expressed in mg/g; * 95% TI: 95% Tolerance Index based on the Population.

**Table 9:** Characterization of phytochemicals present in optimized olive leaf extract (OE) by LC-HRMS analysis in negative and positive modes.

Pk. No.	RT (min)	[M-H]^−^ *m*/*z* Observed	[M-H]^+^ *m*/*z* Observed	∆ ppm	Predicted Molecular Formula	MS/MS *m*/*z*	Compound Identity	Quantification mg/g
1	1.39	181.0717		−0.42	C_6_H_14_O_6_	163, 119, 101, 89 (100), 71, 59	Sugar alcohol	nq
2	1.48	341.1087		−0.77	C_12_H_22_O_11_	179, 119, 89 (100), 71, 59	Gentiobiose	nq
3	1.63	191.0561		0.05	C_7_H_12_O_6_	127, 85	Quinic acid *	6.181 ± 0.104
4	1.86	133.0144		0.73	C_4_H_6_O_5_	115 (100), 89, 71	Malic acid *	6.175 ± 0.104
5	5.90	153.0558		0.45	C_8_H_10_O_3_	123 (100), 95	3-Hydroxytyrosol *	2.328 ± 0.044
6	6.47	315.1085		−0.06	C_14_H_20_O_8_	153 (100), 135, 123, 89, 59	Hydroxytyrosol glucoside	37.510 ± 0.417
7	6.72		229.0706	−0.20	C_10_H_12_O_6_	226, 169 (100), 155, 137, 123, 95	Unknown	nq
8	6.73	389.1087		−0.59	C_16_H_22_O_11_	183, 165, 121, 89, 59 (100)	Oleoside type	nq
9	7.69	137.0609		0.69	C_8_H_10_O_2_	131, 120, 98, 92, 69, 66 (100), 41	Tyrosol *	0.354 ± 0.028
10	9.21	389.1087		−0.59	C_16_H_22_O_11_	345, 203, 121, 89, 69, 59 (100)	Oleoside type	nq
11	9.23		151.0390	−0.10	C_8_H_6_O_3_	N.F.	Unknown	nq
12	9.54	403.1244		−0.48	C_17_H_24_O_11_	203, 121, 101, 89, 71, 59 (100)	Oleoside methylester	nq
13	9.55		165.0545	−0.52	C_9_H_8_O_3_	147, 129, 111, 99, 85, 69 (100)	Unknown	nq
14	9.98	519.1721		0.2	C_22_H_32_O_14_	295, 235, 193 (100), 175,89	Globularitol	nq
15	10.25	377.1451		−0.47	C_16_H_26_O_10_	197 (100), 153	Acyclodihydro-elenolic acid hexoside derivative	nq
16	10.48		234.2063	3.48	C_16_H_25_O	186	Unknown	nq
17	10.64	461.1664		−0.15	C_20_H_30_O_12_	226, 203, 191, 149, 131, 101, 89 (100), 71, 59	Unknown	nq
18	10.86		165.0546	−0.33	C_9_H_8_O_3_	147, 129, 111, 99, 85, 69 (100)	Unknown	nq
19	10.90	403.1244		−0.48	C_17_H_24_O_11_	203, 165, 139, 121 (100), 101, 89, 71, 59	Elenolic acid glucoside	nq
20	11.20	555.1719		−0.10	C_25_H_32_O_14_	323, 223, 203, 151 (100), 89	Hydroxy-oleuropein	1.373 ± 0.004
21	11.41	525.1613		−0.09	C_24_H_30_O_13_	249, 209, 165, 121, 89, 69, 59 (100)	Demethyl-oleuropein	3.605 ± 0.040
22	11.60		611.1607	0.07	C_27_H_32_O_16_	449, 287 (100)	Rutin isomer	nq
23	11.82	623.1982		1.82	C_29_H_36_O_15_	461, 161 (100)	Verbascoside *	2.144 ± 0.011
24	11.91	403.1972		−0.45	C_19_H_32_O_9_	223 (100), 201, 101, 89, 71, 59	2-(2-ethyl-3-hydroxy-6- propionylcyclohexyl) acetic acid glucoside	nq
25	13.48	539.1769		−0.14	C_25_H_32_O_13_	307, 275, 226, 203, 149, 139, 121, 101, 95, 89, 71, 59 (100)	Oleuropein *	143.144 ± 4.914
26	13.49		137.0596	−0.43	C_8_H_8_O_2_	119, 91 (100)	Dehydro-tyrosol	nq
27	14.18	539.1770		−0.02	C_25_H_32_O_13_	465, 307, 275, 203, 149, 101, 95, 89, 85, 71, 59 (100)	Oleuropein type	10.918 ± 0.323
28	14.36	583.2032		0.03	C_27_H_36_O_14_	203, 195, 121, 89, 59 (100)	Unknown	nq
29	14.46		463.1236	0.17	C_22_H_22_O_11_	395, 301 (100), 173	Unknown	nq
30	14.64		379.1388	0.13	C_19_H_22_O_8_	137 (100)	Unknown	nq
31	14.65	539.1769		−0.14	C_25_H_32_O_13_	371, 307, 275, 226, 203 (100), 139, 121, 95, 59	Oleuropein type	37.405 ± 0.008
32	14.85		449.1078	−0.04	C_21_H_20_O_11_	287 (100), 179	Unknown	nq
33	15.47		449.1080	0.43	C_21_H_20_O_11_	287 (100), 176, 128, 85, 61	Unknown	nq
34	16.86		290.2690	2.51	C_20_H_34_O	242 (100), 122, 74	Unknown	nq
35	19.06		323.1465	3.60	C_17_H_23_O_6_	226	Unknown	nq
36	20.36		330.3366	−0.05	C_20_H_43_O_2_N	312 (100), 106, 88	*N*,*N*-dimethyl-safingol	nq
37	23.30		277.2162	0.04	C_18_H_28_O_2_	209, 199 (100), 181, 171, 69	Unknown	nq
38	24.28		233.1934	0.15	C_16_H_24_O	226, 183, 143, 85, 71, 65, 57	Unknown	nq
39	25.66	471.3479		−0.18	C_30_H_48_O_4_	203, 84, 63	Maslinic acid *	1.392 ± 0.029
40	27.27	455.3530		−0.22	C_30_H_48_O_3_	203 (100)	Ursolic acid *	1.089 ± 0.023

* Quantities of the detected compounds were determined using commercial standards; N.F. = not fragmented; nq = not quantified.

**Table 10:** Characteristics of empty and optimized olive leaf extract (OE) Eu-liposomes *vs.* uncoated liposomes.

	MD nm ± SD	PI ± SD	ZP mV ± SD	EE Oleuropein % ± SD	EE Hydroxytyrosol % ± SD
**Empty liposomes**	74 ± 3.0	0.27 ± 0.01	+8 ± 2.1		
**OE liposomes**	73 ± 2.3	0.27 ± 0.01	+7 ± 1.8	62 ± 0.9	96 ± 0.8
**Empty Eu-liposomes**	* 105 ± 10.1	* 0.47 ± 0.01	* −17 ± 3.5		
**OE Eu-liposomes**	° 101 ± 6.1	° 0.46 ± 0.02	° −17 ± 3.5	65 ± 2.1	96 ± 0.3

MD: Mean Diameter; PI: Polydispersity Index; ZP: Zeta Potential; EE: Entrapment Efficiency; each value represents the mean ± standard deviation (SD; *n* > 10); * *p* < 0.001 *vs.* empty liposomes; ° *p* < 0.001 *vs.* OE liposomes.

**Table 11:** Characteristics of empty and optimized *O. europaea* extract (OE) uncoated liposomes *vs.* Eu-liposomes during storage at 4 °C.

	Time (Days)	MD nm ± SD	PI ± SD	ZP mV ± SD
**Empty** **uncoated liposomes**	t_0_	74 ± 3.0	0.27 ± 0.01	+8 ± 2.1
	t_30_	73 ± 6.0	0.27 ± 0.02	+8 ± 2.3
	t_60_	77 ± 3.4	0.29 ± 0.04	+9 ± 2.0
	t_90_	77 ± 7.4	0.31 ± 0.05	+10 ± 5.1
	t_120_	73 ± 5.8	0.26 ± 0.02	+8 ± 2.3
	t_150_	79 ± 8.3	0.32 ± 0.05	+12 ± 3.5
**OE** **uncoated** **liposomes**	t_0_	73 ± 2.3	0.27 ± 0.01	+7 ± 1.8
	t_30_	72 ± 0.9	0.25 ± 0.01	+8 ± 2.6
	t_60_	79 ± 8.3	0.32 ± 0.07	+7 ± 2.9
	t_90_	79 ± 7.1	0.31 ± 0.02	+14 ± 2.8
	t_120_	79 ± 9.6	0.31 ± 0.06	+12 ± 3.8
	t_150_	75 ± 2.3	0.30 ± 0.04	+14 ± 3.8
**Empty** **Eu-liposomes**	t_0_	105 ± 10.1	0.47 ± 0.01	−17 ± 3.5
	t_30_	96 ± 3.1	0.42 ± 0.01	−18 ± 7.8
	t_60_	94 ± 4.0	0.40 ± 0.02	−18 ± 6.2
	t_90_	94 ± 2.7	0.39 ± 0.02	−15 ± 0.8
	t_120_	93 ± 1.8	0.39 ± 0.01	−15 ± 3.4
	t_150_	93 ± 3.5	0.38 ± 0.01	−15 ± 0.3
**OE** **Eu-liposomes**	t_0_	101 ± 6.1	0.46 ± 0.02	−17 ± 3.5
	t_30_	94.0± 2.1	0.40 ± 0.02	−22 ± 2.7
	t_60_	94 ± 2.7	0.39 ± 0.03	−24 ± 4.5
	t_90_	93 ± 1.9	0.39 ± 0.02	−17 ± 2.0
	t_120_	93 ± 2.4	0.39 ± 0.01	−17 ± 2.7
	t_150_	92 ± 1.5	0.38 ± 0.01	−15 ± 3.3

MD: Mean Diameter; PI: Polydispersity Index; ZP: Zeta Potential; each value represents the mean ± standard deviation (SD; *n* = 4).

**Table 12:** Olive leaf extract (OE) uncoated liposomes *vs.* Eu-liposomes in USP simulated gastric and intestinal fluids.

	Simulated Fluid	MD nm ± SD	PI ± SD	ZP mV ± SD
**OE** **uncoated liposomest_2h_**	SGF	83 ± 1.3	0.28 ± 0.02	+4 ± 2.2
**OE Eu-liposomes t_2h_**		105 ± 14.3	0.20 ± 0.02	−9 ± 5.2
**OE** **uncoated liposomes t_6h_**	SIF	84 ± 2.0	0.28 ± 0.03	−38 ± 3.6
**OE Eu-liposomes t_6h_**		100 ± 13.0	0.32 ± 0.09	−25 ± 6.0

MD: Mean Diameter; PI: Polydispersity Index; ZP: Zeta Potential; SGF: USP Simulated Gastric Fluid; SIF: USP Simulated Intestinal Fluid; each value represents the mean ± standard deviation (SD; *n* = 4).

## Data Availability

The original contributions presented in this study are included in the article. Further inquiries can be directed to the corresponding authors.

## Supplementary Materials

The following supporting information can be downloaded at https://www.mdpi.com/article/10.3390/pharmaceutics18080965/s1, Table S1: Extraction yield (%) obtained for each experimental run of the Box–Behnken design and for the experimentally validated optimized extract; Figure S1: UHPLC-HRMS chromatograms of quinic acid in the optimized olive leaf extract (A) and quinic acid standard (B), together with the corresponding mass spectrum (C) acquired in negative ion mode; Figure S2: UHPLC-HRMS chromatograms of malic acid in the optimized olive leaf extract (A) and malic acid standard (B), together with the corresponding mass spectrum (C) acquired in negative ion mode; Figure S3: UHPLC-HRMS chromatograms of 3-hydroxytyrosol in the optimized olive leaf extract (A) and 3-hydroxytyrosol standard (B), together with the corresponding mass spectrum (C) acquired in negative ion mode; Figure S4: UHPLC-HRMS chromatograms of tyrosol in the optimized olive leaf extract (A) and tyrosol standard (B), together with the corresponding mass spectrum (C) acquired in negative ion mode; Figure S5: UHPLC-HRMS chromatograms of verbascoside in the optimized olive leaf extract (A) and verbascoside standard (B), together with the corresponding mass spectrum (C) acquired in negative ion mode; Figure S6: UHPLC-HRMS chromatograms of oleuropein in the optimized olive leaf extract (A) and oleuropein standard (B), together with the corresponding mass spectrum (C) acquired in negative ion mode; Figure S7: UHPLC-HRMS chromatograms of maslinic acid in the optimized olive leaf extract (A) and maslinic acid standard (B), together with the corresponding mass spectrum (C) acquired in negative ion mode; Figure S8: UHPLC-HRMS chromatograms of ursolic acid in the optimized olive leaf extract (A) and ursolic acid standard (B), together with the corresponding mass spectrum (C) acquired in negative ion mode.

## Abbreviations

The following abbreviations are used in this manuscript:

2-NBDG	2-(*N*-(7-Nitrobenz-2-oxa-1,3-diazol-4-yl)amino)-2-deoxyglucose
BBD	Box–Behnken Design
DCFH-DA	2′,7′-Dichlorodihydrofluorescein diacetate
DOTAP	1,2-Dioleoyloxy-3-trimethylammonium propane chloride
DPPH	2,2-Diphenyl-1-picrylhydrazyl
FRAP	Ferric Reducing Antioxidant Power
MD	Mean Diameter
mg GAE/g	milligrams of Gallic Acid Equivalents per gram of dry extract
mg TE/g	milligrams of Trolox Equivalents per gram of dry extract
MTT	3-[4,5-dimethylthiazol-2-yl]-2,5 diphenyl tetrazolium bromide
NAC	*N*-acetyl-L-cysteine
OE	Optimized olive leaf extract
OE Eu-LIP	Optimized olive leaf extract in Eu-liposomes
OE sol	Optimized olive leaf extract in solution
P90G	Phospholipon^®^ 90G
PBS	Phosphate Buffered Saline
PI	Polydispersity Index
RSM	Response Surface Methodology
SGF	Simulated Gastric Fluid
SIF	Simulated Intestinal Fluid
T2DM	Type 2 diabetes mellitus
TPC	Total Phenolic Content
TPTZ	2,4,6-Tripyridyl-*s*-triazina
Trolox	6-Hydroxy-2,5,7,8-tetramethylchroman-2-carboxylic acid
USP	United States Pharmacopoeia
ZP	Zeta Potential
