# Supplementary material for: Liposomal Delivery of Olea europaea L. Leaf Polyphenols: From Extraction to Functional Evaluation in a Hyperglycemia Cell Model
Source: Pharmaceutics. 2026 Aug 6;18(8):965. doi: 10.3390/pharmaceutics18080965 (PMC13516623; doi:10.3390/pharmaceutics18080965)
Supplement: Supplementary file 1 [file pharmaceutics-18-00965-s001.zip › pharmaceutics-4435830-supplementary.pdf]

pharmaceutics

Supplementary material

Liposomal Delivery of *Olea europaea* L. Leaf Polyphenols: From Extraction to Functional Evaluation in a Hyperglycemia Cell Model

Immacolata Faraone [1,†], Maria Ponticelli [1,†], Simona Demuro [2], Antonio Vassallo [1,*], Margherita Accardo [1,*], Ludovica Lela [1], Carla Caddeo [2,‡] and Luigi Milella [1,‡]

[1] Department of Health Sciences, University of Basilicata, Via dell'Ateneo Lucano 10, 85100 Potenza, Italy; immacolata.faraone@unibas.it (I.F.); maria.ponticelli@unibas.it (M.P.); ludovica.lela@unibas.it (L.L.); luigi.milella@unibas.it (L.M.)
[2] Department of Life and Environmental Sciences, University of Cagliari, 09042 Monserrato, Cagliari, Italy; simona.demuro@unica.it (S.D.); caddeoc@unica.it (C.C.)
* Correspondence: antonio.vassallo@unibas.it (A.V.); margherita.accardo@unibas.it (M.A.); Tel.: +39-0971-205-624 (A.V.)
† These authors contributed equally to this work.
‡ These authors contributed equally to this work.

Index

Table S1. Extraction yield (%) obtained for each experimental run of the Box–Behnken design and for the experimentally validated optimized extract.

Figure S1. UHPLC-HRMS chromatograms of quinic acid in the optimized olive leaf extract (**A**) and quinic acid standard (**B**), together with the corresponding mass spectrum (**C**) acquired in negative ion mode.

Figure S2. UHPLC-HRMS chromatograms of malic acid in the optimized olive leaf extract (**A**) and malic acid standard (**B**), together with the corresponding mass spectrum (**C**) acquired in negative ion mode.

Figure S3. UHPLC-HRMS chromatograms of 3-hydroxytyrosol in the optimized olive leaf extract (**A**) and 3-hydroxytyrosol standard (**B**), together with the corresponding mass spectrum (**C**) acquired in negative ion mode.

Figure S4. UHPLC-HRMS chromatograms of tyrosol in the optimized olive leaf extract (**A**) and tyrosol standard (**B**), together with the corresponding mass spectrum (**C**) acquired in negative ion mode.

Figure S5. UHPLC-HRMS chromatograms of verbascoside in the optimized olive leaf extract (**A**) and verbascoside standard (**B**), together with the corresponding mass spectrum (**C**) acquired in negative ion mode.

Figure S6. UHPLC-HRMS chromatograms of oleuropein in the optimized olive leaf extract (**A**) and oleuropein standard (**B**), together with the corresponding mass spectrum (**C**) acquired in negative ion mode.

Figure S7. UHPLC-HRMS chromatograms of maslinic acid in the optimized olive leaf extract (**A**) and maslinic acid standard (**B**), together with the corresponding mass spectrum (**C**) acquired in negative ion mode.

Figure S8. UHPLC-HRMS chromatograms of ursolic acid in the optimized olive leaf extract (**A**) and ursolic acid standard (**B**), together with the corresponding mass spectrum (**C**) acquired in negative ion mode.

Table S1. Extraction yield (%) obtained for each experimental run of the Box–Behnken design and for the experimentally validated optimized extract.

Run	Extraction yield (%)
**1**	5.15
**2**	9.52
**3**	10.81
**4**	15.84
**5**	8.75
**6**	16.24
**7**	7.29
**8**	39.16
**9**	16.79
**10**	42.90
**11**	17.08
**12**	19.89
**13**	28.46
**14**	16.58
**15**	15.97
**16**	12.54
**17**	20.82
**Optimized extract**	37.95

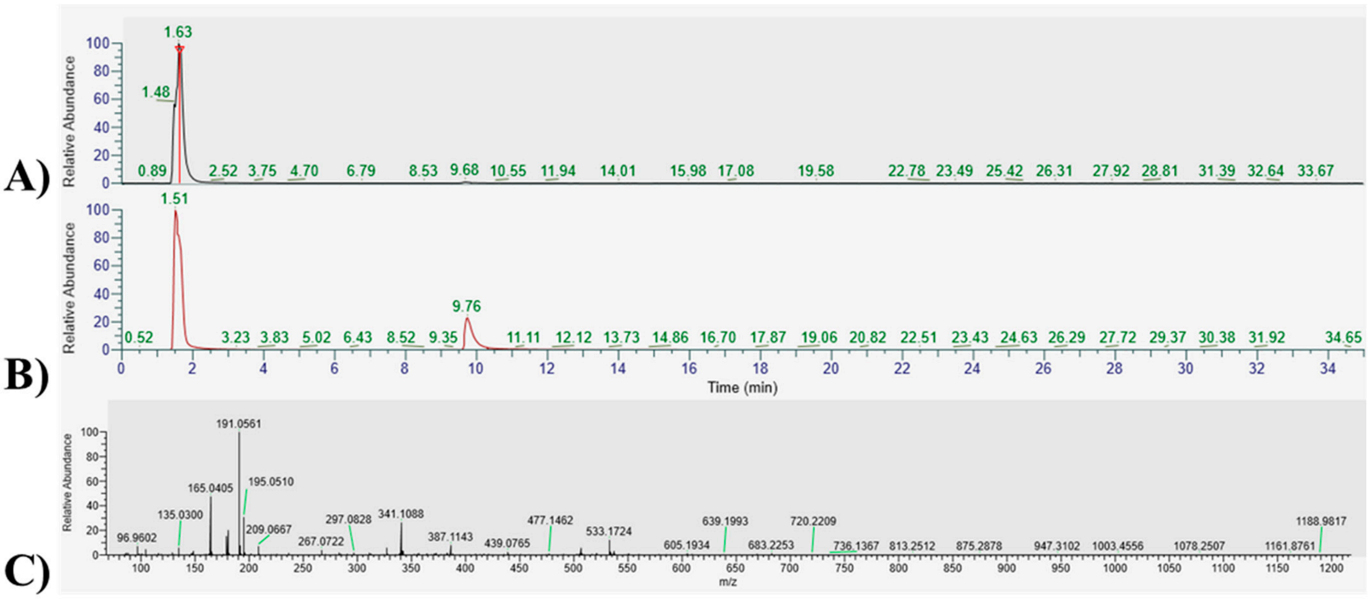

Figure S1. UHPLC-HRMS chromatograms of quinic acid in the optimized olive leaf extract (**A**) and quinic acid standard (**B**), together with the corresponding mass spectrum (**C**) acquired in negative ion mode.

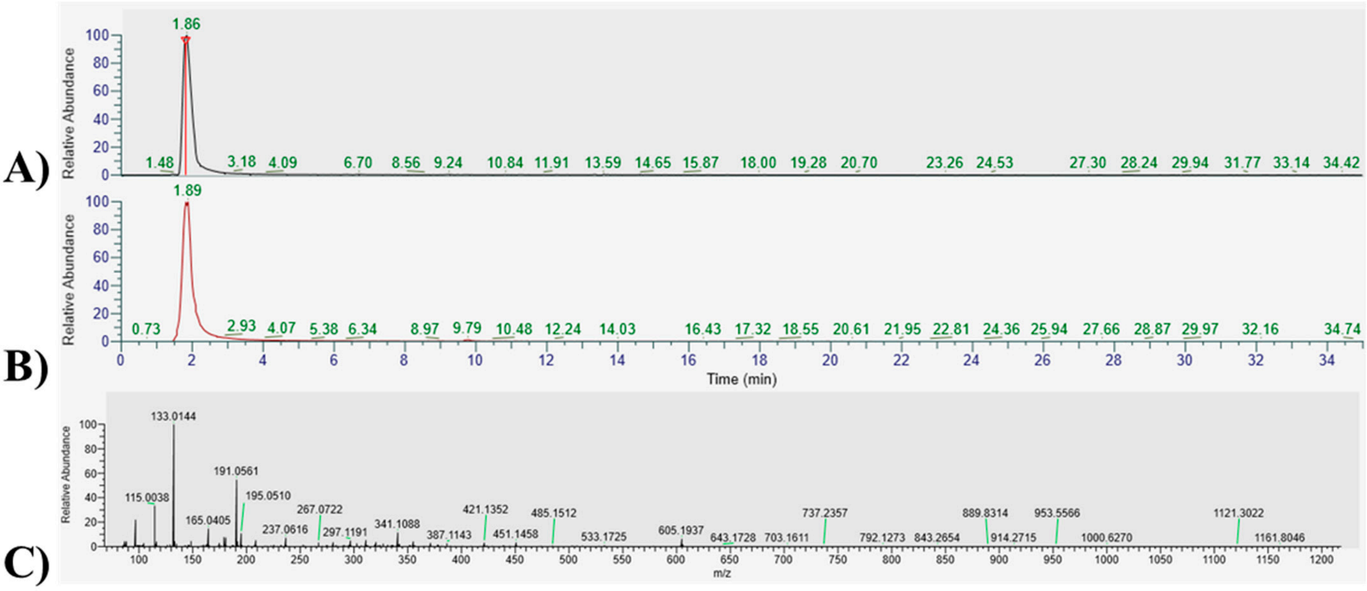

Figure S2. UHPLC-HRMS chromatograms of malic acid in the optimized olive leaf extract (**A**) and malic acid standard (**B**), together with the corresponding mass spectrum (**C**) acquired in negative ion mode.

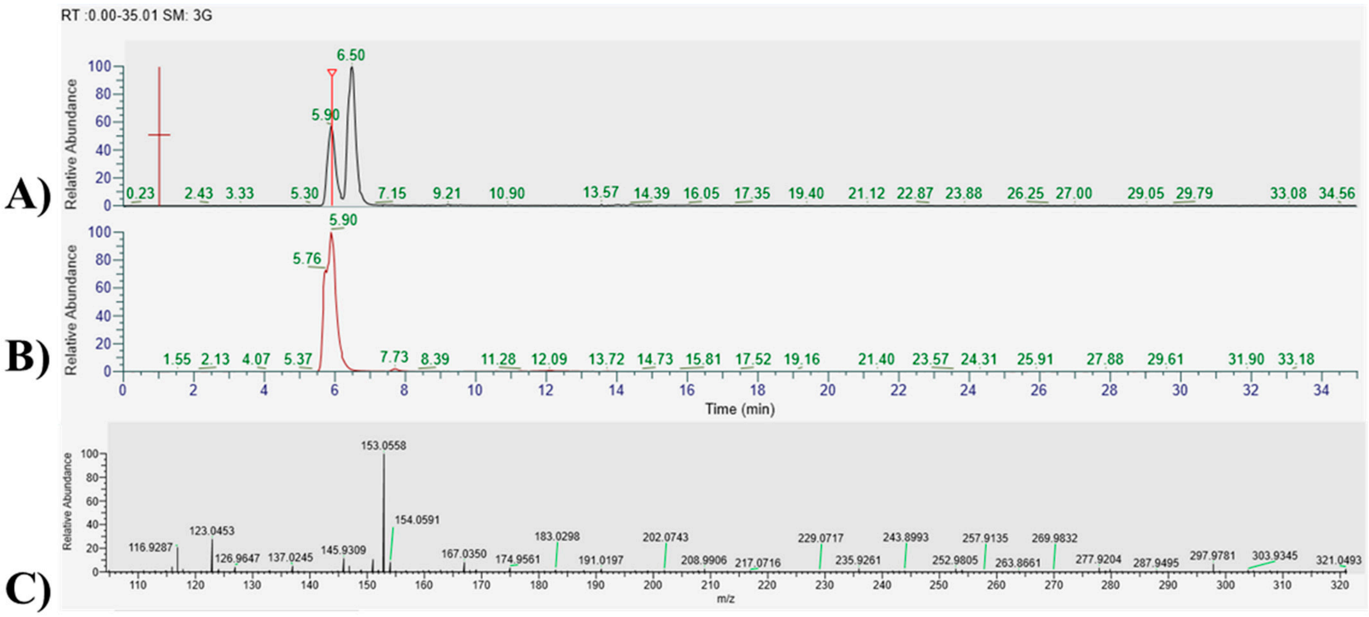

Figure S3. UHPLC-HRMS chromatograms of 3-hydroxytyrosol in the optimized olive leaf extract (**A**) and 3-hydroxytyrosol standard (**B**), together with the corresponding mass spectrum (**C**) acquired in negative ion mode.

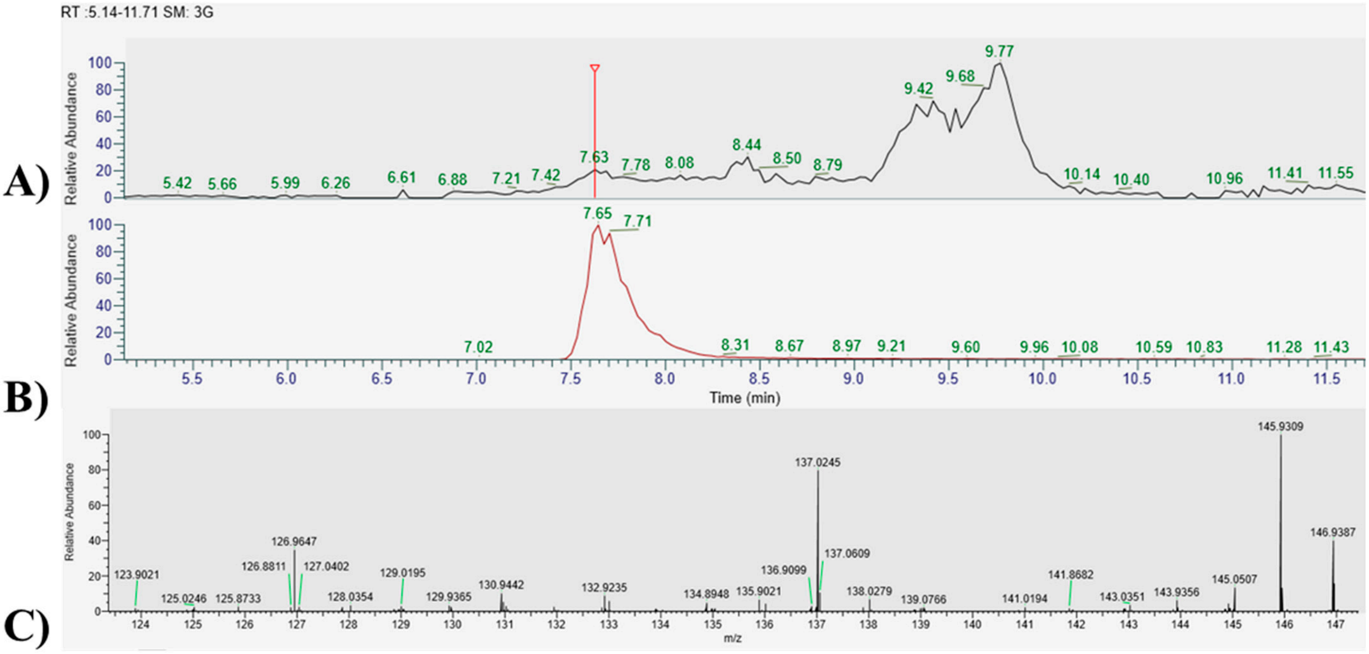

Figure S4. UHPLC-HRMS chromatograms of tyrosol in the optimized olive leaf extract (**A**) and tyrosol standard (**B**), together with the corresponding mass spectrum (**C**) acquired in negative ion mode.

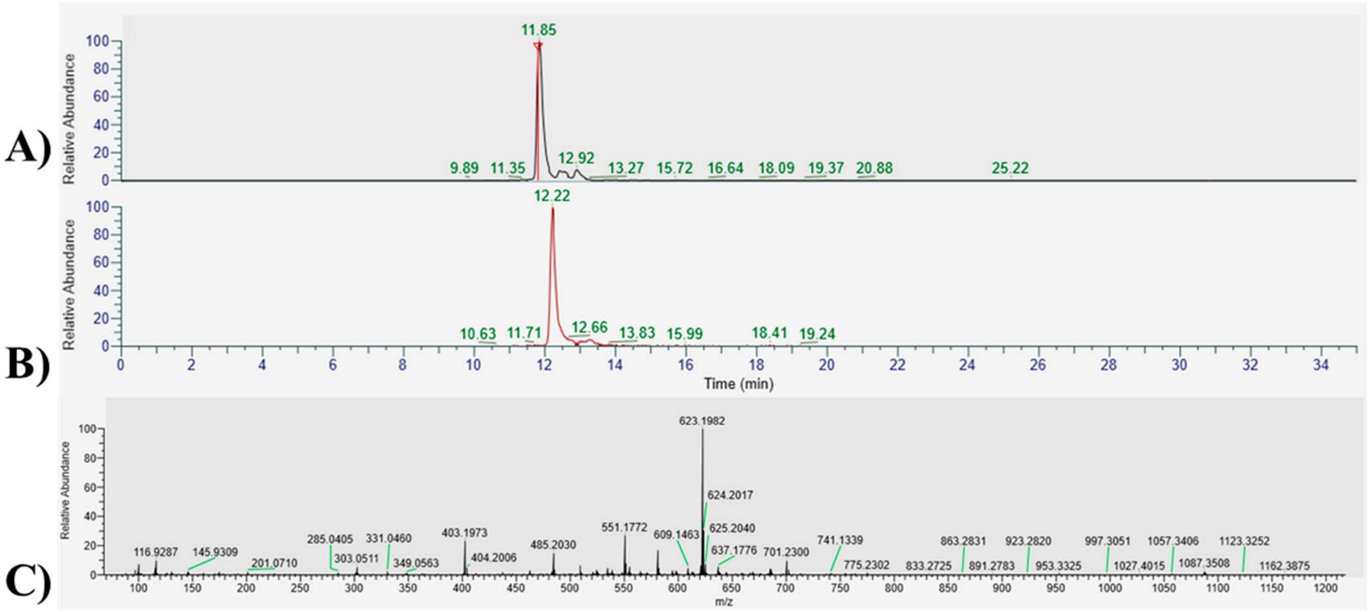

Figure S5. UHPLC-HRMS chromatograms of verbascoside in the optimized olive leaf extract (**A**) and verbascoside standard (**B**), together with the corresponding mass spectrum (**C**) acquired in negative ion mode.

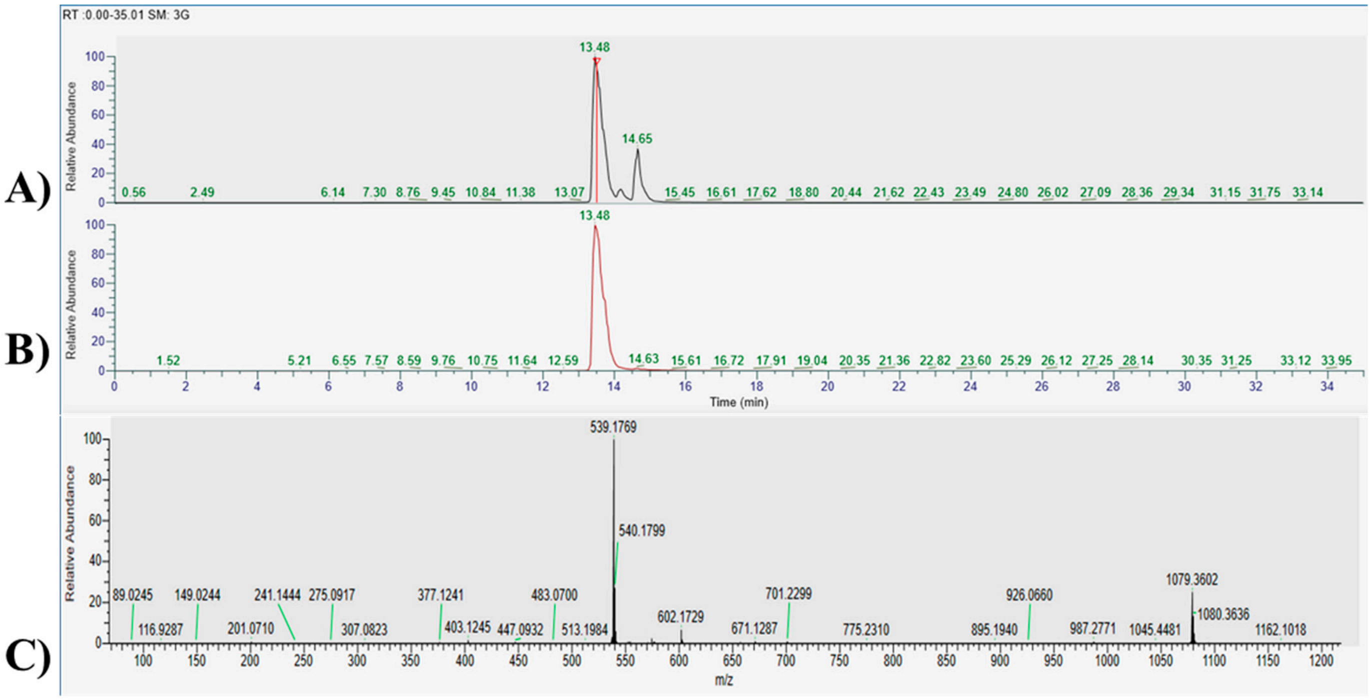

Figure S6. UHPLC-HRMS chromatograms of oleuropein in the optimized olive leaf extract (**A**) and oleuropein standard (**B**), together with the corresponding mass spectrum (**C**) acquired in negative ion mode.

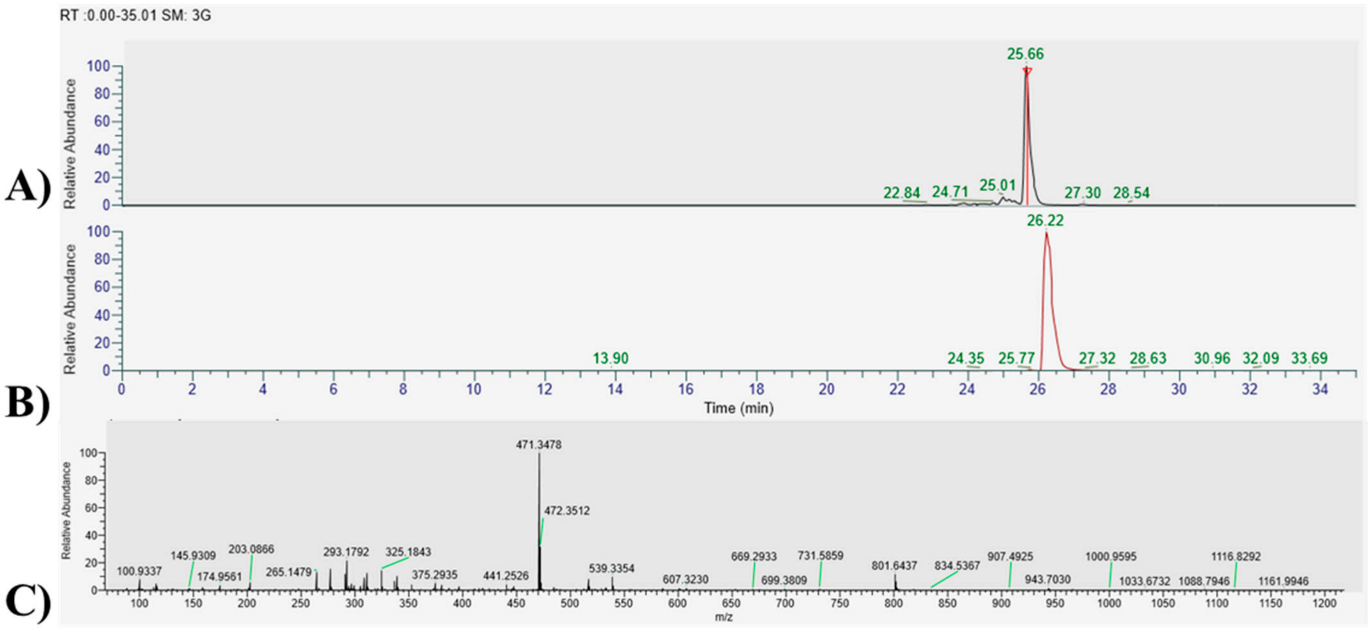

Figure S7. UHPLC-HRMS chromatograms of maslinic acid in the optimized olive leaf extract (**A**) and maslinic acid standard (**B**), together with the corresponding mass spectrum (**C**) acquired in negative ion mode.

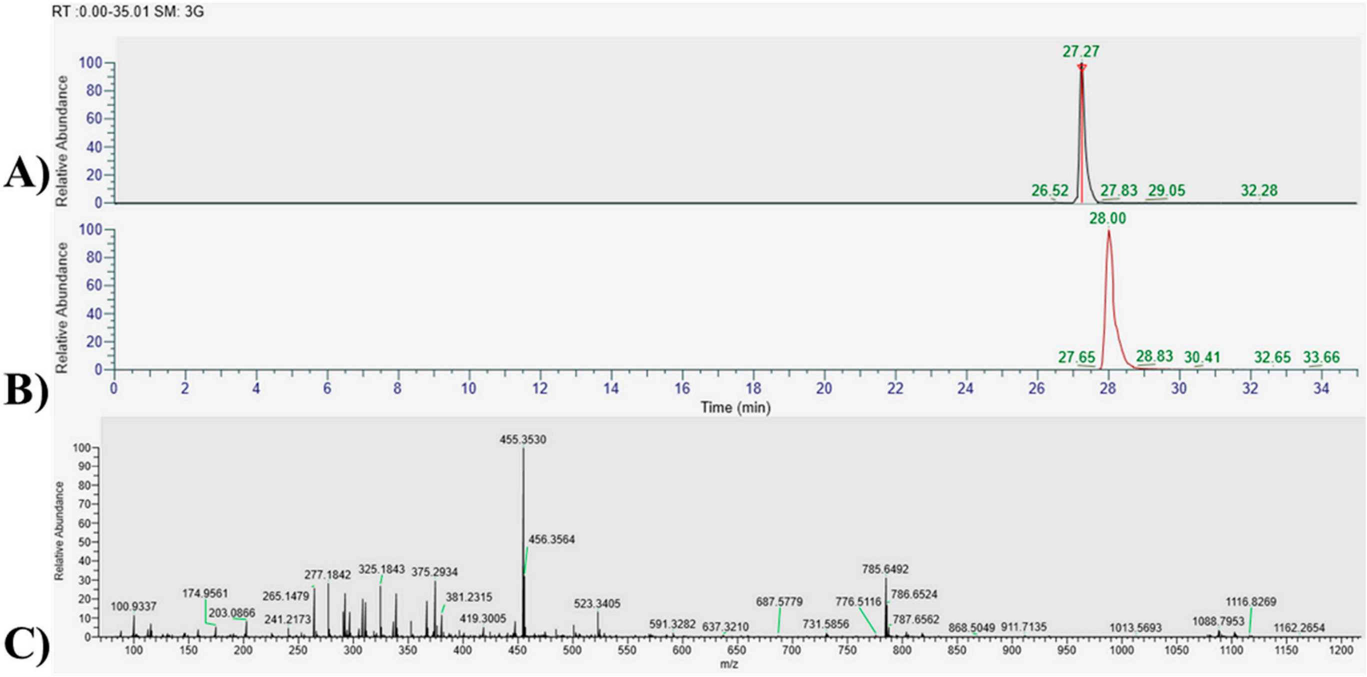

Figure S8. UHPLC-HRMS chromatograms of ursolic acid in the optimized olive leaf extract (**A**) and ursolic acid standard (**B**), together with the corresponding mass spectrum (**C**) acquired in negative ion mode.
